# Default mode network electrophysiological dynamics and causal role in creative thinking

**DOI:** 10.1093/brain/awae199

**Published:** 2024-06-18

**Authors:** Eleonora Bartoli, Ethan Devara, Huy Q Dang, Rikki Rabinovich, Raissa K Mathura, Adrish Anand, Bailey R Pascuzzi, Joshua Adkinson, Yoed N Kenett, Kelly R Bijanki, Sameer A Sheth, Ben Shofty

**Affiliations:** Department of Neurosurgery, Baylor College of Medicine, Houston, TX 77030, USA; Department of Neurosurgery, Baylor College of Medicine, Houston, TX 77030, USA; Department of Neurosurgery, Baylor College of Medicine, Houston, TX 77030, USA; Department of Neurosurgery, Clinical Neuroscience Center, University of Utah, Salt Lake City, UT 84132, USA; Department of Neurosurgery, Baylor College of Medicine, Houston, TX 77030, USA; Department of Neurosurgery, Baylor College of Medicine, Houston, TX 77030, USA; Department of Neurosurgery, Baylor College of Medicine, Houston, TX 77030, USA; Department of Neurosurgery, Baylor College of Medicine, Houston, TX 77030, USA; Faculty of Data and Decision Sciences, Technion—Israel Institute of Technology, Haifa, 3200003Israel; Department of Neurosurgery, Baylor College of Medicine, Houston, TX 77030, USA; Department of Neuroscience, Baylor College of Medicine, Houston, TX 77030, USA; Department of Neurosurgery, Baylor College of Medicine, Houston, TX 77030, USA; Department of Neuroscience, Baylor College of Medicine, Houston, TX 77030, USA; Department of Neurosurgery, Clinical Neuroscience Center, University of Utah, Salt Lake City, UT 84132, USA

**Keywords:** default mode network, intracranial recordings, brain stimulation, divergent thought, creativity, mind wandering

## Abstract

The default mode network (DMN) is a widely distributed, intrinsic brain network thought to play a crucial role in internally directed cognition. The present study employs stereo-EEG in 13 human patients, obtaining high resolution neural recordings across multiple canonical DMN regions during two processes that have been associated with creative thinking: spontaneous and divergent thought. We probe these two DMN-associated higher cognitive functions through mind wandering and alternate uses tasks, respectively. Our results reveal DMN recruitment during both tasks, as well as a task-specific dissociation in spatiotemporal response dynamics.

When compared to the fronto-parietal network, DMN activity was characterized by a stronger increase in gamma band power (30–70 Hz) coupled with lower theta band power (4–8 Hz). The difference in activity between the two networks was especially strong during the mind wandering task. Within the DMN, we found that the tasks showed different dynamics, with the alternate uses task engaging the DMN more during the initial stage of the task, and mind wandering in the later stage. Gamma power changes were mainly driven by lateral DMN sites, while theta power displayed task-specific effects. During alternate uses task, theta changes did not show spatial differences within the DMN, while mind wandering was associated to an early lateral and late dorsomedial DMN engagement. Furthermore, causal manipulations of DMN regions using direct cortical stimulation preferentially decreased the originality of responses in the alternative uses task, without affecting fluency or mind wandering.

Our results suggest that DMN activity is flexibly modulated as a function of specific cognitive processes and supports its causal role in divergent thinking. These findings shed light on the neural constructs supporting different forms of cognition and provide causal evidence for the role of DMN in the generation of original connections among concepts.

## Teaser

The default mode network is a widely distributed, intrinsic brain network thought to play a crucial role in internally directed cognition. Bartoli *et al.* reveal the temporal dynamics of the default mode network and show that its activity helps generate original connections between concepts.

**See Lopez-Persem *et al.* (https://doi.org/10.1093/brain/awae294) for a scientific commentary on this article.**

## Introduction

Internal mentation and other introspective thought processes underlie the stream of human consciousness. The default mode network (DMN), a fundamental neurobiological system, is a dispersed cortical network that is thought to underlie spontaneous cognitive processes.^1^ While prominent during periods of rumination or even at rest, the DMN deactivates when attention is directed to the external world and towards other cognitively demanding tasks.^2,3^ The discovery of DMN using functional MRI (fMRI) has led to a plethora of functional imaging-based studies correlating DMN activity with various cognitive functions and disease states.^4-6^ Some of these studies have emphasized activity within the DMN, investigating activation during cognitive processes, such as episodic memory retrieval, future simulation and mind wandering, while others have looked at DMN deactivation in response to externally directed tasks in the form of visual search or mental arithmetics.^7-10^

Despite the proliferation of neuroimaging studies that have queried the human DMN over the past 20 years, there still exists a relative paucity of information regarding the electrophysiological underpinnings of the network.^6^ Most of the pioneering studies on the electrophysiological basis of the DMN have been focused on individual hubs due to the network's dispersed spatial structure. Earlier studies looking into intracranial electrophysiology of the DMN addressed mechanistic network properties and their correlation with previous fMRI blood oxygen level-dependent (BOLD) findings, providing essential evidence matching fMRI activation and deactivation with massive neuronal firing patterns as identified using local field potentials.^11^ Most of these studies have centred on investigations into high-frequency neural signals, known to be a robust correlate of fMRI BOLD signals and, to a given extent, spiking activity in the brain.^12,13^

Localized deactivations in high-frequency activity in default mode regions, such as the posterior cingulate cortex (PCC), ventrolateral and rostromedial prefrontal cortex have been noted on a variety of externally focused tasks, including mental arithmetic, visual search and reading.^14-19^ Conversely, task-induced elevations in high-frequency activity within the DMN have been discussed in the context of internally directed cognition, reported in areas such as the PCC and retrosplenial cortex in tasks of self-referential judgement and autobiographical memory.^20-23^ More recent investigations have started to unravel the correspondence of inter- and intra-network dynamics with cognitive functions. For example, mentalizing about self and others recruits the DMN with a specific posterior-to-anterior spatiotemporal pattern.^24^ Within DMN areas, correlated slow fluctuations (<4 Hz) occur during rest, memory encoding and recall, while cross-network interactions seem to rely on higher frequency band signals.^25^ These types of intra-network slow fluctuations at rest have been shown to modify firing rates and gamma band (>30 Hz) local field potentials^26^ and are a reliable correlate of BOLD signal spontaneous fluctuations.^27^ Inter-network interactions have been reported in the beta (13–20 Hz) and in the theta range (4–8 Hz)^20,25^ during memory encoding/recall and autobiographical memory retrieval, respectively.

Despite the substantial amount of evidence indicating the involvement and interaction of the DMN with other networks during various complex cognitive functions, there is very limited causal evidence. A large sample study employing direct stimulation to interfere with posteromedial cortex activity at rest, did not produce any notable effects.^28^ However, a recent study demonstrated a causal link between divergent thinking and DMN integrity using individualized direct cortical stimulation during task performance.^29^ Disrupting the network caused a reduction in the number of responses during an alternate uses task (i.e. requiring formulation of non-canonical uses for everyday objects). The distinctive role of DMN in internal mental manipulation and simulation^30,31^ further implies its contribution to divergent thinking. From this perspective, divergent thinking is a high order DMN-associated function^32-35^ that involves retrieving and combining several different types of information to generate a novel and useful idea.^36,37^

**Figure 1:**
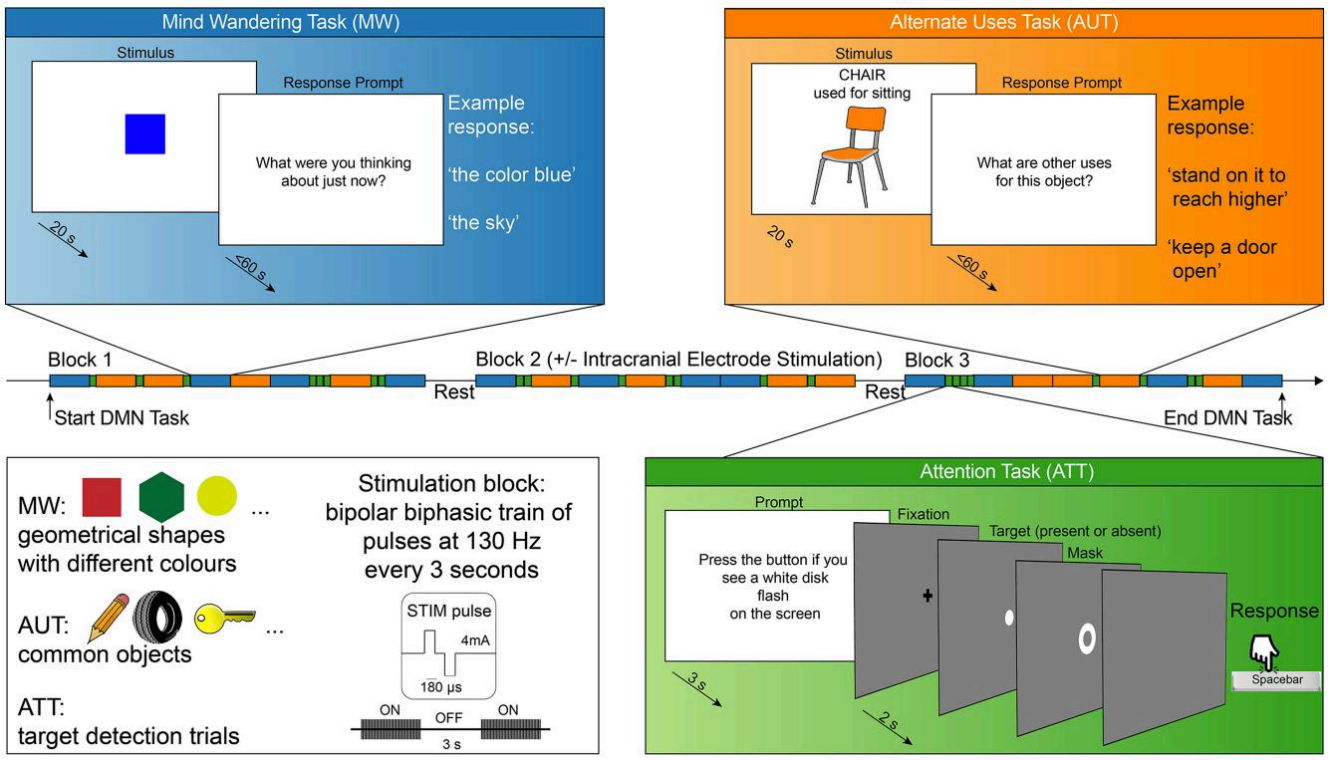
**Experimental task design**. Each box shows a schematic of one of the three tasks: mind wandering (MW), alternate uses (AUT) and sustained attention (ATT), plus an overview of the stimuli and the stimulation parameters (*bottom left*). In the *middle*, a schematic depicts the subdivision of the experiments in three blocks, with stimulation being delivered during the second block. The MW and AUT trials had a stimulus presentation stage and a response stage. During the stimulus stage, a visual cue instructs to let the mind freely wander (MW) or think about alternative uses for the displayed object (AUT). During the response stage, the participant was probed to verbalize either the train of thoughts that just occurred (MW) or to list the alternative uses for the item just displayed (AUT). In the sustained attention task, the subject was cued to detect the brief presentation of a white disk, which could appear alone (target only), followed by a white ring mask (target plus mask) or not appear at all (mask only). Images depicting the AUT items were obtained from: https://www.clker.com/.

**Figure 2:**
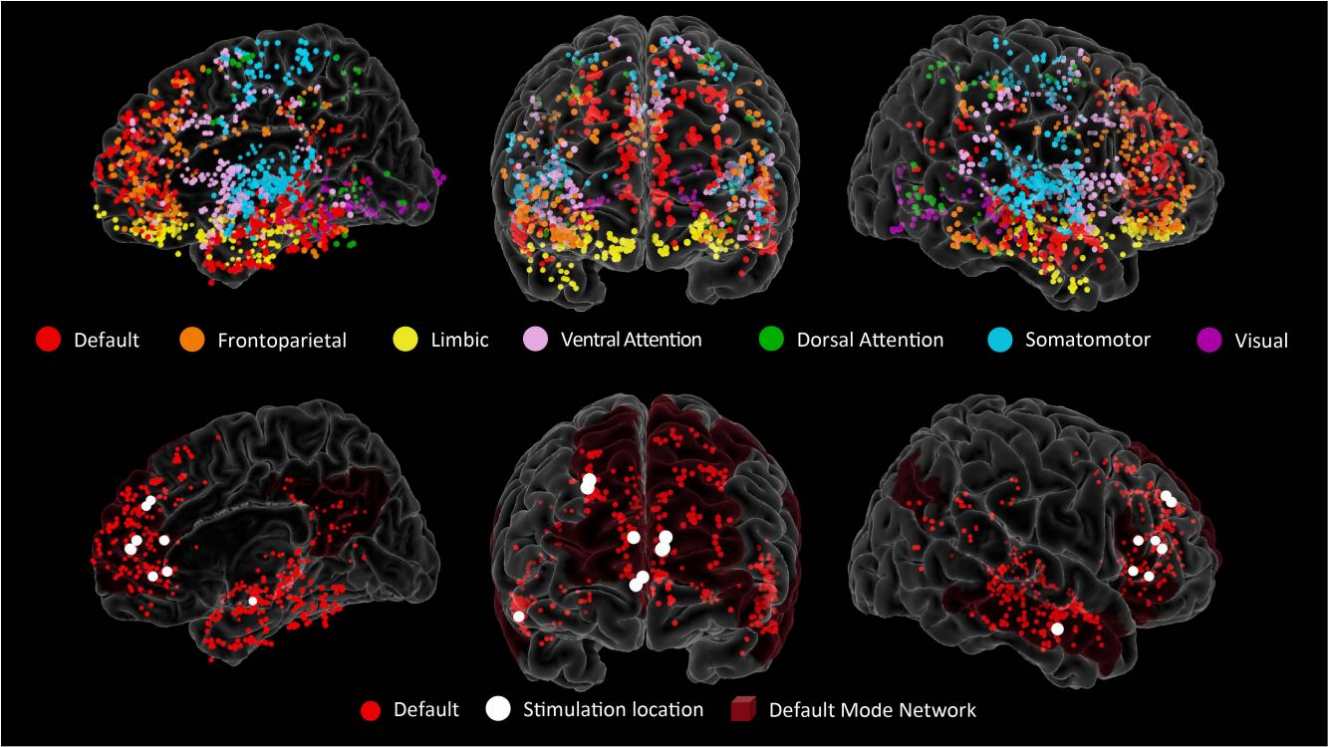
**Electrode locations**. The *top row* shows all the intracranial electrode locations (*n* = 13 subjects) represented as spheres on a template brain, colour-coded by the Yeo 7 network Atlas assignment based on their proximity to the cortical surface.^38^ The *bottom row* shows only the electrodes in the default mode network (DMN; red) and the location of the stimulation contacts (white). The stimulation electrodes are represented as a larger sphere centred between the pair of electrodes used for bipolar stimulation, *n* = 9 subjects: one right middle temporal gyrus, two right superior frontal gyrus, three right anterior cingulate, three left anterior cingulate (note that two of the three locations in the left anterior cingulate are almost completely overlapping).

Though significant strides have been made, we have yet to assess the full span of spatiotemporal dynamics occurring within the DMN as a function of ongoing cognitive processes. The current study aims to investigate (i) how the DMN reorganizes and modifies its activity dynamically based on different modes of thought; (ii) what parts of the network are flexibly recruited; and (iii) the causal role of DMN during divergent thinking. To this end, we assess DMN-associated thought processes through mind wandering and alternate uses tasks (AUT), as well as a control sustained attention task (Fig. 1) in a sample of 13 patients undergoing monitoring with intracranial electrodes (stereo-EEG, sEEG). Taking advantage of the distributed spatial coverage and high temporal resolution of sEEG recordings, we were able to record neural activity within the canonical DMN precisely during these cognitive tasks (Fig. 2) and compare it to fronto-parietal control (FPN) network activity. As signals of interest, we focus on gamma and theta range signals, believed to capture local and long range activity, respectively. In addition, we leveraged electrical high frequency stimulation to disrupt DMN and test the causal role of the DMN in cognitive thought processes. Through these experiments, we link unique patterns of theta and gamma range signals to different stages of mind wandering and divergent thinking. In addition, we demonstrate that disrupting DMN activity reduces originality without affecting mind wandering.

## Materials and methods

### Human subjects

Thirteen subjects (seven males, six females, mean age of 41 years, ranging from 19–60 years) consented to participate in this study while they underwent invasive epilepsy monitoring with sEEG at Baylor St. Luke's Medical Center (Houston, Texas, USA). Detailed subject information is reported in Table 1. Experimental procedures were approved by the Institution Review Board at Baylor College of Medicine (IRB protocol number H-18112). No patients with prior surgical resection in the areas of interest participated in this study. Experiments were recorded while interictal epileptic discharges were absent in the areas of interest. Stimulation was performed when deemed safe by the clinical team supervising the experiment and while the patients were on their typical antiepileptic medication regime (Supplementary material, Section 1.1 and Supplementary Table 1).

**Table 1:** Sample information

Subject	Age	Sex	Electrodes in dorsomedial-DMN	Electrodes in lateral temporal-DMN	Total electrodes in DMN	Total electrodes in FPN	Stimulation experiment	Stimulated locations	MNI coordinates
1	47	M	14	13	27	9	y	R ACC	(5.5, 53, −5)
2	19	M	8	27	35	3	N	–	–
3	57	M	8	8	16	5	y	L ACC	(−5.5, 67.5, −9)
4	24	M	16	21	37	15	y	L ACC	(−5, 67, −9.5)
5	52	F	16	–	16	2	y	R ACC	(2, 51, −20.5)
6	53	F	–	9	9	11	n	–	–
7	25	F	11	11	22	18	y	R MTG	(59, 10.5, −41)
8	41	F	6	24	30	10	y	R SFG-dlPFC	(22, 65.5, 13)
9	60	M	6	–	6	2	n	–	–
10	44	F	3	20	23	9	y	L ACC	(−6.5, 64, −5)
11	46	F	5	23	28	4	n	–	–
12	42	M	5	19	24	4	y	R SFG-dlPFC	(21.5, 63, 16)
13	25	M	50	10	60	15	y	R ACC	(4.5, 58.5, −23)

For each participant in the study (*n* = 13) the table reports the age at the time of the experiment (years), the sex (M = male, F = female), the number of electrodes in dorso-medial and lateral temporal-DMN and their sum (count), the number of electrodes in the fronto-parietal control network (FPN), whether the subject took part in the stimulation experiment (yes/no), the stimulation location (R = right; L = left; ACC = anterior cingulate; MTG = middle temporal gyrus; SFG-dLPFC = superior frontal gyrus extending into dorso-lateral prefrontal cortex) and the MNI coordinate of the stimulation (midpoint between the pair of electrodes employed for bipolar stimulation). DMN = default mode network.

### Task design

All subjects performed the experimental paradigm while reclined in a hospital bed in a quiet room. All tasks were presented on an adjustable monitor (1920 × 1080 resolution, 47.5 × 26.7 cm screen size, 60 Hz refresh rate, connected to a PC running Windows 10 Pro at a viewing distance of 57 cm, such that 1 cm on the screen corresponds to ∼1° visual angle). Tasks were programmed using Psychtoolbox-3 functions (v3.0.16)^39^ running on MATLAB (R2019a, MathWorks, MA, USA). An experimenter was seated by the bedside throughout the experiment to give subjects instructions. The experiment consisted of three types of tasks (Fig. 1): (i) a mind wandering task; (ii) an alternate uses task (AUT); and (iii) a visual sustained attention task (ATT). During mind wandering, the subjects fixated on a coloured shape in the centre of the screen for 20 s. They were then asked, ‘What were you thinking about just now?’ to which they verbalized their thoughts for as long as they could or until 1 min had passed. The mind wandering task was adapted from the ‘Shape Expectations’ task developed by O’Callaghan and colleagues.^40^ During AUT, the subjects fixated on an object in the centre of the screen for 20 s and were informed of its typical function. They were then asked, ‘What are other uses for this object?’ to which they listed out as many alternate uses that they could conceive or until 1 min had passed. Their responses to the mind wandering and AUT tasks were recorded verbatim into Microsoft Excel (Microsoft, WA, USA). During ATT, the subjects attended to the centre of the screen where a fixation cross would flash (250 ms) to signal the start of the ATT trial: subjects were required to attend the screen for 400 ms and report the detection of a target stimulus (small white disk, subtending 0.5° visual angle) by button press (maximum response time allowed 2 s). The target would briefly flash on 75% of trials (for a single frame, ∼17 ms), either on its own or followed by a white annulus mask (1°, flashed for two frames, with a 50 ms lag between target and mask). On the remaining 25% of trials, only the annulus mask appeared without the target. Button presses were recorded by the experiment software. The sustained attention task was utilized to ensure that any observed DMN changes are not an epiphenomenon that would occur with any task. Between each trial there was a short break (grey screen, 2 s). The experiment was divided into three blocks, each composed of four mind wandering trials, four AUT trials and eight ATT trials (16 total trials per block). For the mind wandering and AUT trials, different coloured shapes and objects were used across all blocks, such that no stimuli were repeated, to avoid learning effects. The stimuli used in each task were identical for all patients and their order was randomized within each block. A subset of patients (*n* = 9) received intracranial stimulation during a portion of the experiment (Block 2).

### Stereo-EEG probes

Reported data were acquired by sEEG depth probes. The sEEG probes had either a 0.8 mm diameter, with 8–16 electrode contacts along the probe with a 3.5 mm centre-to-centre distance (PMT Corporation) or a 1.28 mm diameter, with nine recording contacts, with a 5.0 mm centre-to-centre distance between contacts (AdTech Medical Instrument Corporation).

### Electrode localization and selection

For each subject, we determined electrode locations by employing the software pipeline intracranial Electrode Visualization, iELVis.^41^ In short, the postoperative CT image was registered to the preoperative T_1_ anatomical MRI image using FSL.^42^ The location of each electrode was then identified in the CT-MRI overlay using BioImage Suite.^43^ The electrode anatomical locations were classified based on their proximity to the cortical surface model, reconstructed by the T_1_ image using Freesurfer (version 6.0^44^). The anatomical assignment of each electrode was manually verified by an expert in neuroanatomy (B.S.). We further labelled each electrode by mapping onto each individual cortical surface the 7 Network estimate atlas^38^ and finding the most likely cortical parcellation estimate using a 5 mm radius around each electrode. The atlas consists of seven networks based on intrinsic connectivity (default mode, fronto-parietal, limbic, ventral attention, dorsal attention, somatomotor, visual network). To visualize electrodes of interest on a common brain, we transformed electrode coordinates into the MNI average stereotaxic model and represented them as spheres on the Freesurfer Colin27 brain using Multi-Modality Visualization Tool, MMVT^45^ (Fig. 2). Electrodes classified as recording from the DMN were selected for the main analyses of the current study. For a subset of analyses, we selected electrodes from the FPN and from the somatomotor (see later). Within the DMN, we further identified two subsystems: dorsomedial-DMN (electrodes recording from ventromedial prefrontal cortex, anterior cingulate, superior frontal gyrus, posterior cingulate, posterior parietal, parahippocampal gyrus) and lateral temporal areas, which we labelled lateral-DMN (electrodes recording from middle temporal gyrus, superior and middle temporal sulci). Note that the division into these two subsystems is partially dictated by the cortical sampling availability. In particular, the dorsomedial-DMN system includes frontal, cingulate and parietal contributions and ideally it would have been interesting to isolate each unique contribution, but our sample did not provide enough within-subject cortical coverage across these areas to reliably interpret any subdivision beyond the one presented here. In addition to extracting DMN contacts, we also used the 7 Network estimate atlas to identify electrodes in the FPN. In this atlas, DMN and FPN regions are often in close proximity with interlacing borders. Thus, electrodes near transition zones between the two networks (5 mm radius) were excluded. Further electrode exclusions were applied based on the signal quality, as described later. See Table 1 for a list of all the electrode counts in DMN (and its subsystems) and FPN (see Supplementary material, Section 1.2 and Supplementary Fig. 1 for electrode coverage information at the individual level). Last, we identified electrodes recording from the motor cortex for a control analysis (Supplementary material, Section 2.2).

### Electrophysiological recording and preprocessing

SEEG signals were recorded by a 256 channel BlackRock Cerebus system (BlackRock Microsystems) at 2 kHz sampling rate, with a fourth order Butterworth bandpass filter of 0.3–500 Hz. SEEG recordings were referenced to an electrode contact visually determined to be in white matter. A photoresistor sensor recording at 30 kHz was attached to the task monitor to synchronize intracranial recordings to the stimulus presentation time. All signals were processed in MATLAB (R2019a, MathWorks, MA, USA). Raw sEEG signals were first inspected for line noise, recording artefacts (e.g. contamination by muscle activity, presence of large deflections, additional noise components) and interictal epileptic spikes. Electrodes with artefacts and epileptic spikes were excluded from further analysis. After this exclusion was applied, we obtained 333 total electrodes in the DMN (148 electrodes in dorsomedial-DMN and 185 in lateral-DMN), ranging from 6–60 for each subject. Across our sample, 10 subjects had electrodes recording from both DMN subsystems, two subjects only from dorsomedial-DMN and one subject only from lateral-DMN. In addition, we obtained 107 electrodes in FPN, ranging from 2 to 18 for each subject (Table 1 and Supplementary Fig. 1).

Next, the signal from each electrode was notch filtered (60 Hz and harmonics) and re-referenced to the neighbouring electrodes using a Laplacian local average reference. Finally, re-referenced signals were downsampled to 500 Hz and spectrally decomposed using a family of Morlet wavelets (seven cycles), with centre frequencies ranging logarithmically from 1 to 200 Hz in 100 steps. Frequency band power was obtained by averaging the magnitude of the Morlet wavelet decomposition result between 4–8 Hz for theta and 30–70 Hz for gamma. Power values during each trial were normalized to per cent change with respect to the pre-trial baseline period (500–100 ms before stimulus onset). Power % change values computed for each electrode location were then averaged over trials. Outliers were removed at the trial-level before averaging [values >3 standard deviations (SD) above the mean or if per cent signal change exceeded 1000%]. On average, fewer than two trials were removed per patient/per electrode.

### Cortical stimulation

Bipolar stimulation (trains of biphasic pulses at 130 Hz, with 4 mA amplitude and 180 µs pulse width) was delivered in cycles of 3 s on, 3 s off to a pair of electrode contacts located within one DMN node, using a Cerestim R96 stimulator (BlackRock Microsystems). This stimulation pattern occurred during the entire second block of the experiment (Fig. 1) and was not time-locked to any specific event. The stimulation parameters were selected to resemble deep brain stimulation parameters (130 Hz and 180 μs) while falling within the FDA guidelines for safe stimulation based on charge density (which varies as a function of amplitude, pulse-width and electrode contact surface area). Overall, our stimulation parameters were selected to maximize patient safety (i.e. minimize risk of epileptiform after discharges or seizures). Out of the 13 participants, four participants did not receive stimulation due to clinical factors (i.e. timing considerations, seizure onset zone proximity, etc.). Two patients received stimulation with modified parameters (Subject 1: 50 Hz in place of 130 Hz, other parameters the same; Subject 4: 90 μs pulse width in place of 180 μs, other parameters the same). For each of the nine participants receiving stimulation, a pair of electrode contacts within the DMN network was selected [three in left anterior cingulate cortex (ACC), three in right ACC, one in right middle temporal gyrus (MTG), two in right superior frontal gyrus-dorsolateral prefrontal cortex (SFG-dlPFC)] (Fig. 2 and Table 1). The electrodes were selected based on anatomical and clinical considerations (not part of the suspected seizure onset zone) and were subjected to an established pipeline to evaluate the safety of the stimulation targets and parameters with respect to epileptiform activity.^46^ In brief, the contacts selected for stimulation were evaluated by the attending epileptologist following an established procedure using trains of stimulations at increasing amplitudes up to the target level (here, 4 mA). This procedure is used to exclude from stimulation experiments any electrodes displaying epileptiform after-discharge, or subjective reports of any paraesthesia, mood or sensory changes, or aura-like activity. Last, the experiment was conducted only when the patients were on full dosage anti-epileptic treatment, thus further minimizing the chances of provoking seizures during research stimulation. The electrophysiological data from the stimulation period were excluded from the analysis.

### Behavioural scoring

The AUT and mind wandering tasks were scored based on the semantic or conceptual distance between each object or item (e.g. chair; blue square) and the open-ended responses that the subjects generated. Semantic distance captures the relationship between ideas, concepts or other texts, based on the assumption that words that occur in similar contexts are similar in meaning.^47^ In creativity research, several studies have shown how such a quantitative measure strongly captures subjective ratings of originality of responses.^48,49^ Thus, such a measure can be used to quantify originality. The response data were collated into a Microsoft Excel sheet individually for each patient. Each instance of the original object was listed next to a single corresponding response. Responses indicating an absence of thought (such as ‘I could not think of anything’ or ‘nothing comes to mind’) were not considered meaningful in the context of the task and were therefore removed from the dataset. Scoring the originality of the responses was performed using SemDis,^48^ an automated algorithm to compute semantic distance in creativity research (http://semdis.wlu.psu.edu/). SemDis scores range from 0–2, with increasing scores indicating greater semantic distance. This algorithm combines multiple commonly used methods to vectorize and calculate the cosine distance between resulting vectors, quantifying the meaning of the words in multiple semantic spaces. This process results in a more accurate representation of the relationship between the words/phrases than any individual method. Each subject's behavioural data were uploaded into the SemDis website, using the following steps: (i) remove filler words (e.g. a, the, and), leaving only the words that represent the true meaning of the phrase; (ii) clean the data, removing numbers, symbols and other special characters that cannot be vectorized; (iii) use a multiplicative compositional model; (iv) calculate the SemDis scores in all five available semantic spaces to fully capture various meanings of both the cues and responses; and (v) use the average score across all semantic spaces to compute the mean SemDis score for each item.

For further behavioural analysis (to score variability and flexibility of responses), we used the spaCy python library^50^ to perform the following steps. We first cleaned the phrases by removing punctuation and stop words (spaCy defaults, as well as the words ‘use’, ‘things’ and ‘stuff’). We then vectorized the phrases using the Universal Sentence Encoder text embedding model.^51^ To calculate variability between a set of responses for a given item, we determined the semantic distance between each pair of responses. Notably, unlike the originality score above, this analysis does not account for the identity of the cue (item) itself, nor the distance between it and the responses. We quantified the relationship between each pair of responses as the cosine distance between the two response vectors; the sum of the distances for all response pairs (total cosine distance) was used as the measure of variability. To investigate how responses clustered into discrete categories, we used Python's scikit-learn software^52^ to perform t-distributed stochastic neighbour embedding (t-SNE) clustering (parameters: perplexity = 5; learning rate = 1) on all patients’ responses for each item. As a result of this dimensionality reduction process, we were able to visualize the responses in two dimensions. Subsequently, we used the scikit-learn affinity propagation clustering algorithm to identify semantic categories.

Finally, the ATT task served to track whether the subjects were engaged and paying attention to the screen. Button presses (corresponding to participants reporting a target detection) were tabulated by the experiment software and stored in a behavioural file. The probability of reporting a target (hit rate) was higher for target-only trials than for trials with the target followed by the mask (70% and 43%), the false alarm rate (reporting a target during mask-only trials) was 20%. This pattern confirmed that brief duration of the target and the backward masking made the attentional task challenging, and the 70% hit rate to target-only trials confirmed that the participants were engaged and paying attention. The task also served as a control for the specificity of the results for default versus attentional cognitive domains (Supplementary Fig. 2A).

### Statistical analyses

Statistical analyses focused on signals in the theta (4–8 Hz) and gamma frequency ranges (30–70 Hz) after baseline normalization as described earlier. Primarily, multi-level mixed effects models were adopted to account for the unbalanced and nested data structure in the current study (i.e. multiple electrodes from each subject): experimental conditions were set as fixed effects, while subjects and electrodes were set as nested random effects. This approach accounts for the non-independence between observations (e.g. different electrodes from the same subject) while preserving the richness of the dataset (e.g. avoiding averaging across electrodes). In practice, to evaluate the importance of a fixed effect (e.g. task type) the model was compared to a simpler model without the coefficient of interest but with identical random effect structure using chi-square difference tests. This approach tests if the more complex model is significantly better at capturing the data (i.e. if the added parameters yield to a significant reduction of the residuals). *Post hoc* differences were evaluated by using *z*-statistic approximation with two-sided probability and adjusting *P*-values for multiple comparison using Bonferroni (indicated by *P*-adj in the results unless otherwise specified). Exact *P*-values are reported for values >0.001 (any smaller *P*-values are simplified as *P* < 0.001). All analyses were performed in R^53^ using lmer^54^ and multcomp packages.

#### Effects of interest

As detailed previously, the tasks comprised two stages: a stimulus presentation stage, termed ‘Stimulus’ and a patient response stage, termed ‘Response’. We computed theta and gamma power during the first 15 s from the onset of each stage (employing a sliding 1 s window with 50% overlap, leading to 29 time bins centred 500 ms apart, starting at 500 ms from the onset of each stage; e.g. the first time bin is centred at 500 ms from the onset and computed by averaging the data from 0–1 s, the second is centred at 1 s computed over 0.5–1.5, etc.). The 15-s window was chosen to give ample time to capture the cognitive processes involved during both the stimulus and response stages of the AUT and mind wandering tasks. Choosing an appropriate window duration was important as during the response stage the time of active response production/verbalization varied, lasting on average 32 s (25% percentile: 18 s, 75%: 45 s). Based on the distribution of the verbal response durations, we selected 15 s as a compromise to capture meaningful events during both stages across trials and patients. We employed ‘task stage’ (‘Stimulus’, ‘Response’) and ‘time bin’ (29 bins spanning from 500 ms to 15 s) as fixed effects in most of our analyses; depending on the specific analysis, other fixed effects were included in the models, e.g. network (DMN and FPN), task type (AUT, mind wandering), DMN subsystem (dorsomedial, lateral).

#### DMN versus FPN

To compare neural signatures of the DMN and the FPN, we used a linear mixed effects model for each frequency range of interest (theta and gamma) and each task (AUT and mind wandering). The model captured variations in band power as a function of the task stage (Stimulus, Response) and time bin (0 to 15 s in 500 ms steps), both of which were modelled as fixed effects. Subjects and electrodes were modelled as nested random effects. We compared this model to a more complex model that included the fixed effect of network (DMN, FPN) to evaluate the statistical significance of the network effect. The analyses were repeated using a simplified approach (averaging across electrodes for each subject and performing a repeated-measures ANOVA) to confirm the stability of the results across different statistical approaches (Supplementary material, Section 2.1 and Supplementary Tables 2 and 3).

#### DMN dynamics during mind wandering and AUT tasks

We probed further into DMN dynamics during the two default mode tasks, AUT and mind wandering (MW). Variations in band power were investigated with linear mixed effects models for theta and gamma, designating task type (AUT, MW), task stage (Stimulus, Response), time bin (0 to 15 s in 500 ms steps) and DMN subsystems (dorsomedial DMN, lateral DMN) as fixed effects, subjects and electrodes as nested random effects. The importance of each effect and interaction coefficient was assessed using sequential model comparisons (starting from a model with only task type as a fixed effect and adding the effect of task stage, the interaction between the two effects, etc.) as described earlier.

#### Interplay between neural signatures and DMN nodes

To evaluate the interaction between theta and gamma power dynamics across the dorsomedial and lateral DMN nodes and their similarity during the two tasks, we employed hierarchical clustering. This analysis was restricted to a subset of patients with electrodes in both DMN nodes (*n* = 10). The variables for this analysis are theta and gamma power for each task and DMN node. First, we built the set of observations for each variable by extracting the vector containing the power dynamics (0–15 s in 500 ms steps) for both task stages (Stimulus and Response) for each subject (averaging across electrodes recording from the same DMN node within subject), obtaining values corresponding to 29 time points by two task stages for each subject. We then concatenated these observations for all subjects in a long vector, leading to 580 observations for each variable (29 time points by two task stages by 10 subjects). We included the overall theta and gamma power (averaged across DMN nodes and tasks) as two additional variables to identify the major contributors to the overall DMN engagement. The distance between the variables (theta and gamma for each task type and DMN-node combination, plus overall theta and gamma) were computed using 1-correlation values between the 580 observations for each variable. Low distance values between variables indicate that the observations display similar neural signal patterns; high distance values indicate differential/unique patterns. The clustering was performed using complete-linkage and the reliability of the clusters statistically tested with bootstrap resampling (*n* = 10 000) using pvclust and corrplot packages. Bootstrap probability (probability to obtain the same cluster over the resampling iterations) is reported to highlight the robustness of the result.

#### Stimulation effect on behaviour

The mind wandering and AUT originality scores for all subjects taking part in the stimulation portion of the experiment (*n* = 9) were computed for all stimuli (coloured shape or object) and analysed according to stimulation status (no stimulation versus stimulation). The SemDis scores are bound between 0 and 2, with higher values representing higher semantic distance between the stimulus and the response, a proxy of originality. Given the non-normality of the SemDis score, we employed non-parametric testing (Wilcoxon Signed Rank Test, using subject as a pairing variable) to test for the effect of stimulation status on the trial-averaged SemDis scores for both AUT and mind wandering (i.e. averaging the sematic distance scores within stimulation status for each patient). The same approach was employed for other behavioural score metrics (variability, flexibility). For any metric showing a difference related to stimulation status, we further tested the effect across the experimental blocks (Block 1: before stimulation; Block 2: during stimulation; Block 3: after stimulation) and we performed a control analysis removing the subject that underwent stimulation with a different frequency setting (Subject 1, 50 Hz versus 130 Hz; Supplementary material, Section 2.3).

## Results

### DMN versus FPN

**Figure 3:**
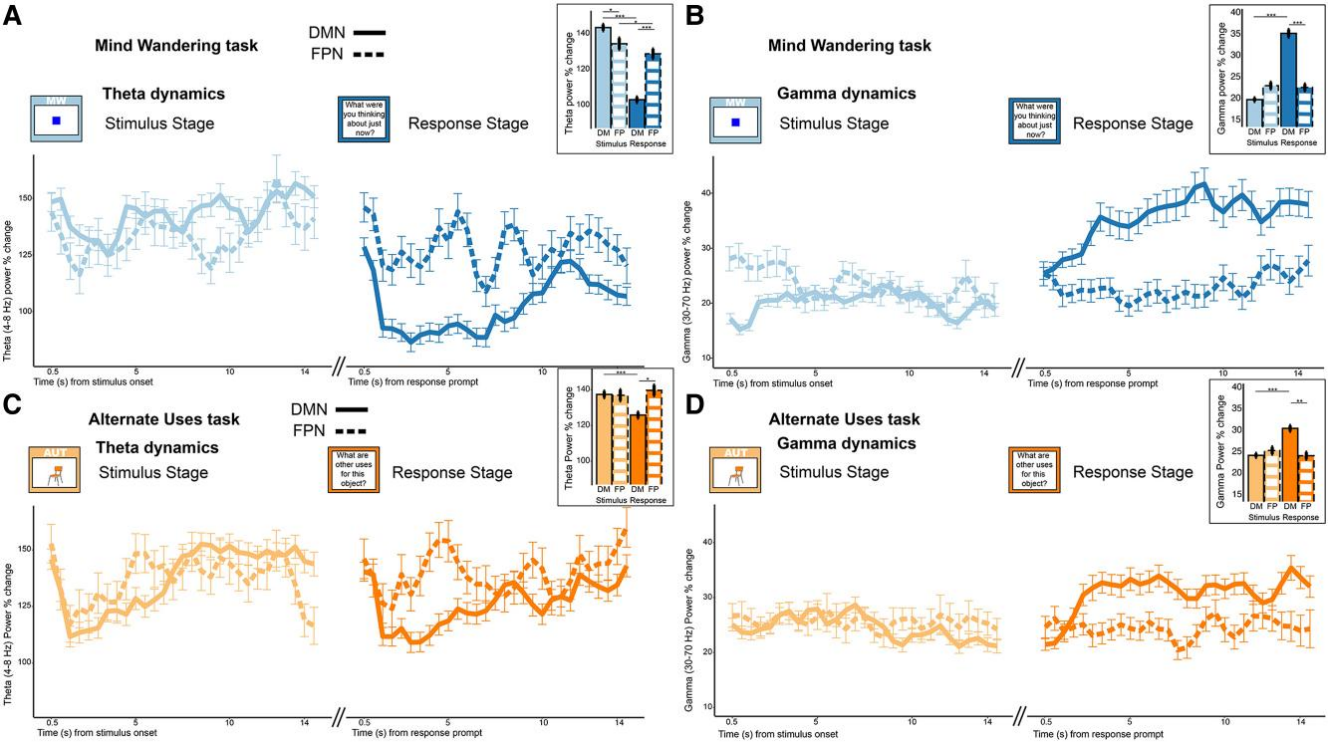
**DMN versus FPN activity during mind wandering and alternate uses tasks**. (**A**): Theta (4–8 Hz, *top*) and (**B**) gamma (30–70 Hz, *bottom*) power modulations over time recorded in default mode network (DMN; solid line) and fronto-parietal control network (FPN; dashed line) during the mind wandering (MW) and (**C** and **D**) alternate uses task (AUT). The dynamics reveal how the DMN is engaged differently from the FPN according to the task and its stage. (**A**) During MW, DMN theta is higher in the stimulus stage and drops to lower values in the response stage, with DMN and FPN being different across both stages. (**B**) DMN gamma power is not different from FPN during the stimulus stage of MW, but it increases significantly during the response stage (i.e. the verbalization of the train of thoughts), while FPN gamma remains stable. (**C**) Theta power is similar between DMN and FPN during the stimulus stage, while a DMN-specific drop in power occurs during the response stage. (**D**) The same pattern emerges for gamma, although in the opposite direction, with a significant increase in DMN gamma specific to the response stage. *Insets* display the *post hoc* comparisons over time-averaged bar plots to aid with visualization, with statistical differences denoted by asterisks (*P-adj < 0.05; **P-adj < 0.01; ***P-adj < 0.001).

The tasks employed in the current study encouraged divergent thinking and mind wanderingprocesses over a long time window. To fully leverage the task design, we investigated the dynamics of theta and gamma oscillations in DMN over a long time course (0–15 s, in 29 time bins), focusing on both the stimulus encoding and the response stage of the tasks (Fig. 3). We first compared the neural activity between the DMN and the FPN for each task separately. The model comparison confirmed that the network was an important effect for both theta and gamma during mind wandering and during AUT (all model comparison with/without the fixed effect of network: all *P*-values < 0.001). Next, we report the outcome of *post hoc* comparisons to isolate the presence of specific differences between the DMN and FPN, within each task and task stage.

#### Mind wandering task

Theta power differed between DMN and FPN. Theta power was significantly higher in DMN than FPN during the stimulus stage, but lower during the response stage (MW stimulus: DMN versus FPN *z* = −2.653, *P*-adj = 0.032; MW response: DMN versus FPN *z* = 5.353, *P*-adj < 0.001; Fig. 3A). Interestingly, both networks exhibited strong increases in theta power during the stimulus period; then, during the response stage, theta power was lower relative to the stimulus stage. While both DMN and FPN displayed this activity pattern, the degree of task stage dependence was more dramatic in the DMN (DMN: stimulus versus response *z* = 37.788, *P*-adj < 0.001; FPN: stimulus versus response *z* = 2.984, *P*-adj = 0.011). With regard to gamma power, the difference between networks was instead present only during the response stage, with DMN gamma power being higher than FPN (MW stimulus: DMN versus FPN *z* = 1.171, *P*-adj = 0.97; MW response: DMN versus FPN *z* = −5.183, *P*-adj < 0.001; Fig. 3B). Moreover, DMN gamma power increased during the response period relative to the stimulus stage only in the DMN, while the FPN did not show a modulation between stages (DMN: stimulus versus response *z* = −41.237, *P*-adj < 0.001; FPN: stimulus versus response *z* = 1.743, *P*-adj = 0.32). Thus, the direction of stage and time-dependent modulations for theta and gamma were in opposite directions: theta power was higher during the stimulus period than the response window, while gamma activity exhibited the reverse pattern, with strongest power increases during the response stage.

#### Alternate uses task

For the divergent thinking task, activity in both the gamma and theta bands was similar across DMN and FPN during the stimulus stage but different between the two networks during the response stage, with lower theta and higher gamma in the DMN versus FPN (theta AUT-stimulus: DMN versus FPN *z* = −0.534, *P*-adj = 1; gamma *z* = 0.596, *P*-adj = 1; theta AUT-response: DMN versus FPN *z* = 2.543, *P*-adj = 0.044; gamma *z* = −3.332, *P*-adj = 0.003; Fig. 3C and D). This pattern was further captured by the difference between stimulus and response occurring within the DMN (theta DMN: stimulus versus response *z* = 10.407, *P*-adj < 0.001; gamma: *z* = −19.266, *P*-adj < 0.001) but not the FPN (all effects non-significant).

Overall, these analyses demonstrated that the activity in the DMN and FPN was different during mind wandering, with theta and gamma power having strong differential responses across the two networks. In the alternate uses task, the DMN and FPN had a similar activity pattern during the stimulus stage, diverging later during the response stage. We note that these results were replicated after averaging across electrodes and re-running a simplified version of the analyses: all repeated-measures ANOVA models resulted in a significant interaction between network and task stage. See Supplementary Tables 2 and 3 for a detailed comparison of the *post hoc* results between the mixed effects and the ANOVA models.

### DMN dynamics

For all subsequent analyses, we focused exclusively on the DMN network. To investigate if the DMN is recruited similarly by divergent thinking and mind wandering, we analysed the neural activity of both tasks in the same model, allowing for direct comparisons of the two tasks as well as their dynamics. First, we verified that task type (AUT, MW), response stage (Stimulus, Response) and their interaction were important for both theta and gamma models (sequential model comparisons adding each fixed effect and interaction term: all *P*-values < 0.001; all *post hoc* comparisons *P*-adj < 0.001; average theta power across the 15 s ± standard error for AUT-stimulus: 137.4 ± 4.9%; MW-stimulus:143.2 ± 4.9%; AUT-response: 125.9 ± 4.6%; MW-response: 102.8 ± 4.3%; gamma for AUT-stimulus: 24.5 ± 1.3%; MW-stimulus: 20.1 ± 1.2%; AUT-response: 30.8 ± 1.8%; MW-response: 35.5 ± 2.5%).

Next, we tested for the effect of time. The dynamics were different across tasks and stages, denoted by the importance of the effect of time bin and its interaction with the combinations of the tasks and stages [Fig. 3B; model comparison with/without time bin in theta: c^2^(1) = 248.3, *P* < 0.001; gamma: c^2^(1) = 49.7, *P* < 0.001; model comparison with/without the interaction terms between time bin and the other fixed effects of task type and task stage in theta: c^2^(3) = 31.6, *P* < 0.001; gamma: c^2^(3) = 151.3, *P* < 0.001]. During the stimulus processing stage (lighter coloured solid lines in Fig. 3), there was a larger increase in gamma power for AUT than for mind wandering, sustained for the first 6–8 s. In theta, we saw a similar effect but in the opposite direction, with lower theta power during the first 6–7 s of AUT; after ∼7 s, AUT-related theta climbed back up to the same level as for mind wandering. During the response stage (darker coloured solid lines in Fig. 3), DMN engagement was stronger for mind wandering than for AUT, with lower theta and higher gamma power over the course of the response window (see Supplementary Fig. 2B for a direct visual overlay of AUT and mind wandering dynamics).

### Dorsomedial and lateral DMN locations contribute differently to mind wandering and divergent thinking

In the previous sections, we showed that DMN is recruited in both mind wandering and divergent thought, albeit in different ways. Next, we tested for sub-network specializations. We found that distinct DMN subregions exhibit differential engagement during task execution. In a model comparing lateral and dorsomedial DMN subsystems, the effect of DMN-subsystem identity and its interaction with the other variables (task type, task stage and time) was significant [model comparison with/without DMN-subsystems and their interaction with the other fixed effects in theta: c^2^(8) = 267.03, *P* < 0.001; gamma: c^2^(8) = 392.7, *P* < 0.001]. Given the complexity of the full model (effect of task type, task stage, DMN-subsystem, time and their interactions), we performed *post hoc* comparisons using a simplified model (refitted without the interaction between time and the other variables), and uncovered the following three-way interaction: theta power was differentially modulated across the two DMN subsystems during mind wandering, but not during AUT. We report the results separately by task for simplicity.

#### Mind wandering task

**Figure 4:**
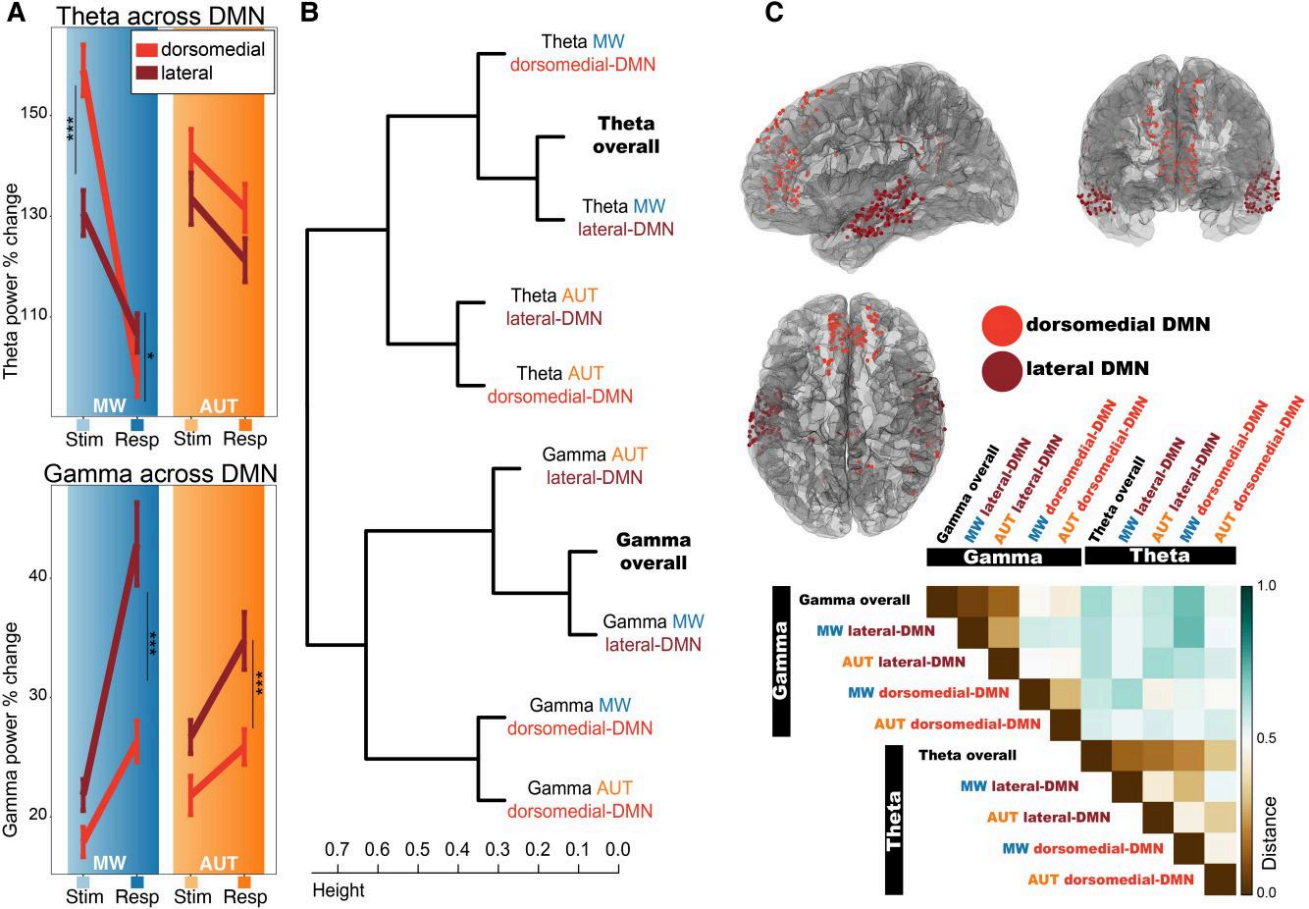
**Interplay between theta and gamma dynamics and spatial contributions within the DMN**. (**A**) Spatial distribution of the task and stage interaction effects within the default mode network (DMN). Theta power differences between dorsomedial and lateral DMN electrode locations are more prominent in mind wandering (MW), while gamma differences are larger in lateral locations with a similar pattern across tasks. Statistical differences based on the *post hoc* comparisons are denoted by asterisks (*P-adj < 0.05; **P-adj < 0.01; ***P-adj < 0.001). (**B**) Hierarchical clustering results obtained by evaluating the distance between the vectors of observations formed by the time course of power values for each stage (stimulus, response) and subject (limiting the analysis to *n* = 10 subjects with electrodes in both DMN subsystems). (**C**) *Top*: Location of electrodes recording from the DMN, colour-coded by the anatomical subdivision in two subsystems. Dorsomedial-DMN includes medial and dorso-lateral frontal-parietal areas (electrodes recording from ventromedial prefrontal cortex, anterior cingulate, superior frontal gyrus, posterior cingulate, postero-medial parietal, parahippocampal gyrus). Lateral-DMN includes lateral temporal areas (electrodes recording from middle temporal gyrus, superior and middle temporal sulci). (**C**) *Bottom*: average distance matrix across variables. (**B** and **C**) *Bottom*: The different direction of theta and gamma modulations during the tasks (first cluster separation in **B**, highest distance values in **C**) and illustrate how the spatio-temporal features of theta and gamma provide non-redundant information about the ongoing cognitive processes. Theta can be used to separate the tasks, while gamma separates the DMN subsystems (second clustering layer: theta separates MW from AUT; gamma separates lateral-DMN from dorsomedial-DMN). In addition, the second clustering layer provides insight on the similarity of the overall gamma and theta effects (averaged across tasks and DMN nodes), suggesting that overall theta is mostly resembling theta during MW, while overall gamma modulations are mostly driven by lateral-DMN. AUT = alternate uses task; Resp = response; Stim = stimulation.

Theta power was higher in dorsomedial DMN (versus lateral DMN) during the mind wandering stimulus stage, but higher in lateral DMN (versus dorsomedial) during the mind wandering response stage (Fig. 4A; lateral versus dorsomedial DMN during MW-stimulus: *z* = −6.6, *P*-adj < 0.001; lateral versus dorsomedial DMN during MW-response: *z* = 2.6, *P*-adj = 0.037). Meanwhile, stimulus-stage gamma power was not significantly different between the two DMN subnetworks; however, response-period gamma power was higher in the lateral DMN (versus dorsomedial) (lateral versus dorsomedial DMN during MW-stimulus: *z* = 1.95, *P*-adj = 0.2; lateral versus dorsomedial DMN during MW-response: *z* = 8.1, *P*-adj < 0.001).

#### Alternate uses task

Theta power did not display any differences across the two DMN nodes for either stage (during AUT-stimulus: *z* = −1.7, *P*-adj = 0.37; during AUT-response: *z* = −2.1, *P*-adj = 0.15). In contrast, gamma power showed a different spatial distribution, trending higher in lateral DMN for both the stimulus and the response stage, with a significant inter-node difference during the response period (during AUT-stimulus: *z* = 2.4, *P*-adj = 0.06; during AUT-response: *z* = 4.4, *P*-adj < 0.001).

The opposite theta modulation in dorsomedial and lateral DMN during the stimulus versus response stages of mind wandering indicates that the process of mind wandering recruits different portions of the DMN depending on the ongoing cognitive process (internalized mind-wandering versus externalized-response production stage). No such dissociation was present in the gamma band, as gamma power was consistently higher in the lateral DMN regions, especially during the response stage, for both tasks. These results are suggestive of sub-specializations within the DMN. To comprehensively unravel the complex interplay between the neural signatures occurring within the DMN for each task type and stage observed so far, we performed a hierarchical clustering analysis based on the similarity of the neural dynamics, described later.

### Interplay between neural signatures and DMN nodes

The degree of similarity between the temporal evolution of theta and gamma activity during each task for the two DMN subsystems was assessed using hierarchical clustering. In this analysis, we also included overall theta and gamma dynamics (regardless of the subsystem or specific task) to help identify the main drivers of the overall neural patterns during divergent thinking and mind wandering (Fig. 4B). The first main separation occurred between theta and gamma: the activity in these two frequency bands displayed opposite patterns of modulation, where lower theta values were paired with enhanced gamma activity [large distance values denoting anticorrelation in Fig. 4C (bottom); electrodes for each DMN subsystem in Fig. 4C (top)]. In addition, the two frequency bands displayed different similarity patterns within their subclusters. Theta power behaved differently for mind wandering and AUT and could be used to separate the two tasks [0.96 bootstrap probability (b.p.)]. Within each task, theta power was similar across the two subsystems (clustered together) (0.86 b.p.). Indeed, theta overall resembled the whole DMN activity during mind wandering and it was clustered with theta during mind wandering for both subsystems (Fig. 4B). Thus, theta was not spatially constrained to a specific subsystem. In contrast, gamma activity differentiated dorsomedial from lateral DMN subsystems (0.95 b.p.). The overall gamma activity was driven by the lateral DMN nodes (1.00 b.p.), regardless of the task (gamma from lateral-DMN during AUT and mind wandering clustered together). This result suggests that gamma dynamics were similar across tasks and mostly driven by lateral regions. Overall, this result demonstrates that theta and gamma play differential, non-redundant roles during mind wandering and divergent thought and display different spatial distribution within the DMN.

### High frequency stimulation of electrodes within the DMN reduces originality of alternate uses

**Figure 5:**
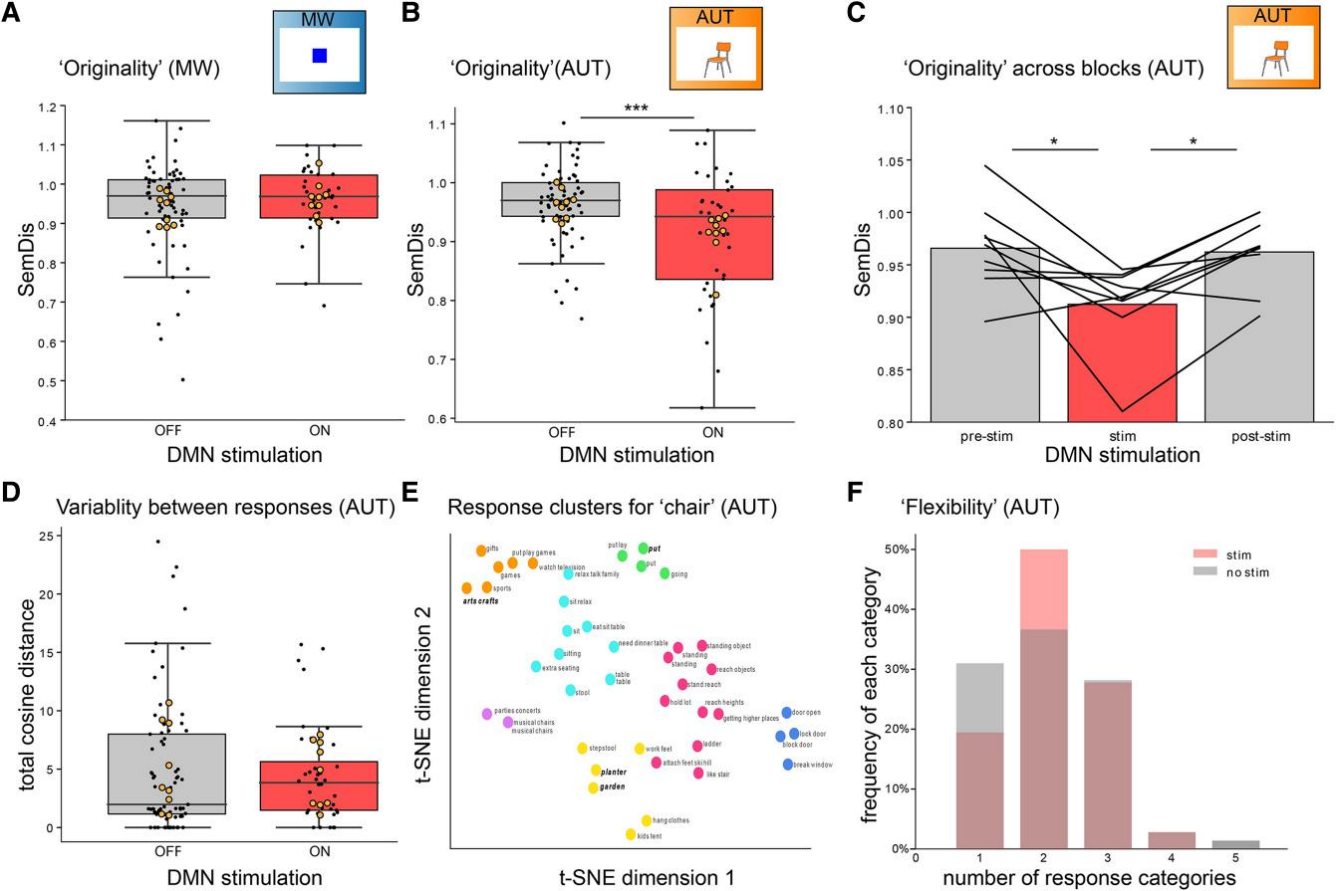
**Stimulation of DMN reduces creativity of responses**. (**A**) Box plots showing SemDis scores (*y*-axis) during mind wandering (MW) as a function of stimulation (stim), showcasing the lack of changes between stimulation on and off during mind wandering (each small black point represents the score for each shape for all nine subjects; each orange dot represents the average score for each subject). (**B**) Same as **A** for alternate uses task (AUT). The SemDis scores, a proxy of the originality of the responses, are reduced during stimulation, indicating a decrease in the originality of the alternate uses responses during high frequency stimulation of the default mode network (DMN). (**C**) Same data as **B** showing the subject-level effect of stimulation across the experimental blocks (each line represents a participant; stimulation was delivered during the second block). (**D**) Total cosine distance, a proxy of variability between the responses, was not affected by stimulation. (As in **A**, black dots in these box plots correspond to all trials; orange dots are the average score per subject) (**E**) Example responses to the item ‘chair’ across the first two t-SNE dimensions, colour-coded by the semantic category clusters. Responses from one participant are highlighted in bold to showcase the number of semantic clusters spanned by that participant. (**F**) Histogram indicating the number of categories into which each participant's responses for each item can be categorized. The number of response clusters, an index of flexibility, was not affected by stimulation. Statistical differences are denoted by asterisks (*P < 0.05; ***P < 0.001). t-SNE = t-distributed stochastic neighbor embedding.

Finally, we assessed whether the creativity of the responses during the mind wandering and AUT was causally dependent on DMN activity. To this end, we manipulated DMN activity by delivering high frequency bipolar stimulation over a neighbouring pair of electrodes located in the DMN. We evaluated the behavioural data from the subjects that received stimulation (*n* = 9) and compared the originality scores for stimulation versus non-stimulation trials (Fig. 5). We averaged scores obtained for stimulation and non-stimulation trials for each subject and performed a non-parametric comparison (with subjects as the pairing variable).

#### Mind wandering task

Stimulation had no effect over mind wandering (SemDis without/with stimulation: 0.97 versus 0.97; W = 28, *P* = 0.3; Fig. 5A).

#### Alternate uses task

Stimulation had the effect of reducing originality in AUT (Fig. 5B; median SemDis without stimulation: 0.97; with stimulation: 0.94; W = 75, *P* < 0.001). To further validate this finding, we performed comparisons between each experimental block to compare SemDis scores before, during and after stimulation (using paired Wilcoxon tests). SemDis scores before versus during stimulation were different (W = 69, *P* = 0.01) as were the scores during versus after stimulation (W = 14, *P* = 0.02). There was no difference in SemDis scores before versus after stimulation (W = 40, *P* = 1). We conclude that stimulation caused a significant reduction in response originality: this effect was specific to stimulation as it did not affect scores for items presented before or after the stimulation block (Fig. 5C). This result was also confirmed after excluding the data from Subject 1 (different stimulation frequency parameter from the rest of the sample) (see Supplementary material, Sections 2.3 and Section 2.4 for an additional control).

Our observation that stimulation during AUT diminished SemDis scores suggests a decrease in the originality of the responses: the responses during stimulation were more semantically similar to the cue word itself. But does DMN stimulation also modulate other aspects of creativity, such as fluency and flexibility? A naïve test of fluency simply counts the number of responses to each presented item. However, multiple similar responses may be ‘original’ (semantically different from the cue word) but virtually identical to each other, so may inflate the ‘fluency’ count for that trial. To address this confound, we devised an alternate metric of fluency that also includes the variability of the responses—consequently, this altered metric of fluency can be called ‘variability’. Specifically, this metric considers the number of responses weighted by how distinct they are from each other, such that very similar responses would not contribute as strongly to this altered metric of fluency (refer to the ‘Materials and methods’ section for details). In our paradigm, stimulation did not affect the variability (Fig. 5D; mean total cosine distance without stimulation: 5.04; with stimulation: 4.59; Wilcoxon signed-rank test, W = 18, *P* = 0.65).

Another facet of creativity is flexibility, which quantifies the number of distinct categories into which the responses fall. To analyse the effect of stimulation on flexibility, we performed t-SNE clustering on the entire set of responses for each item and then used an affinity propagation clustering algorithm to identify semantic categories. Figure 5E shows the outcome of this clustering for one example item (‘chair’). Then, for each patient, we can count the number of clusters from which that patient's responses came. The number of response categories per item ranged from 1–5 (Fig. 5F). For example, the responses of the patient highlighted (bold/italic lettering) in Fig. 5E come from three clusters (even though the patient gave four responses in total). Comparing the number of response categories across patients for trials with and without stimulation yielded no significant results (Wilcoxon signed rank test, W = 21, *P* = 0.91), suggesting no effect of DMN stimulation on flexibility (Fig. 5F).

## Discussion

In this paper, we utilized network-wide intracranial sampling of the DMN and FPN to dissect fast temporal dynamics underlying their functions. We used local field potential (LFP)-derived spectral power changes in theta and gamma bands to differentiate network activation during a DMN-only state (mind wandering) from divergent thinking (AUT), a state in which the DMN should be activated along with other networks. Our goal was to investigate how these different modes of thought engage the DMN. First, we examined the time course of DMN and FPN activation in the alternate uses and mind wandering tasks. We demonstrated network activation during both of our DMN-recruiting tasks, evidenced by stronger modulations in the DMN versus the FPN and characterized by anti-correlated activity between high and low frequency power bands.

When comparing across tasks, we observed an early increase in gamma power in the AUT (versus mind wandering), suggesting that the initial presentation of the object for which the subject needs to find possible alternate uses ‘triggers’ DMN activity^36,55^; in contrast, mind wandering recruited more strongly the DMN during the response stage, indicating that the network is more active during the retrieval and verbalization of the train of thoughts. Overall, mind wandering and AUT exhibited quite similar response profiles, albeit the pattern of lower theta associated with increased gamma was more pronounced in the mind wandering response stage, potentially revealing that the response period in mind wandering more exclusively solicits the DMN, while AUT most likely requires some degree of interaction between DMN and other networks (see also Kenett *et al.*^56^).

Following this general investigation of DMN activity as a whole, we looked into DMN sub-networks and showed that the dorsomedial and lateral areas are differentially engaged by our experimental tasks. Last, we probed the causal relationship between DMN activity and creative thinking and we demonstrated that DMN inhibition through direct cortical stimulation reduces the originality of the responses without impairing fluency, variability or flexibility. In addition, mind wandering was unaffected by stimulation, suggesting that it is either less susceptible to DMN perturbations, or that AUT is more dependent on the DMN ability to synchronize with other networks.

### Differential modulation of theta and gamma-band signals

Neural activity within the DMN was modulated during periods of trial presentation versus pre-trial baseline periods. In general, DMN modulations are characterized by increases in gamma (30–70 Hz) paired with lower theta power (4–8 Hz), specifically for the DMN tasks (AUT and MW) (see Supplementary Fig. 2A for a comparison with ATT and Supplementary Fig. 3 for the association of theta and gamma with other signals). This kind of anti-correlation between low and high frequency is a classic feature of large-scale neural recruitment and network engagement across various systems, related to the balance between excitation and inhibition, and often characterized as a shallower slope of the power spectrum.^57^

### Theta and gamma-band dynamics within the DMN and FPN

To better ascertain how electrophysiological activity in the DMN is specifically modulated throughout mind wandering and divergent thinking, we compared its activity to that of the FPN, a cognitive control network known to act in close association with other functional brain networks, including the DMN and the visual network.^58^ We found that during mind wandering, theta activity differed between the two networks, with higher theta activity in DMN during the early stage and lower theta in DMN during the response stage. This pattern was partially mirrored in the gamma range, where we found the strongest increase in gamma in the DMN (versus FPN) during the response stage (versus stimulus stage). In contrast, during AUT, activity in theta and gamma were closely aligned during the stimulus encoding stage, before branching during the response stage, with lower theta power and higher gamma power in DMN relative to FPN. Additionally, comparing the stimulus stage and the response stage, we saw an evolution in theta and gamma band activity within the DMN, whilst activity in both bands remained similar within the FPN.

Compared to mind wandering, divergent thinking, operationalized via the AUT, exhibited a more unified pattern of activity between the two networks, with neural dynamics being similar amongst the DMN and FPN in both theta and gamma. Theories of creativity and divergent thinking propose the critical importance of both associative and executive processes to develop original ideas.^59^ Divergent thinking requires the individual to search within semantic spaces to form connections among seemingly remote concepts, while executing controlled top-down cognition to parse through inappropriate associations and select for useful, novel thoughts. Prior studies have shown that functional connectivity between the DMN (thought to drive associative processes) and the FPN (supporting executive cognition) has proven a reliable index for an individual's ability for divergent thought.^34,60^ Recent fMRI research is starting to unpack the complex dynamics across these two networks in divergent thinking.^56,61,62^ The similarity in DMN and FPN theta and gamma dynamics during the stimulus phase of the alternate uses task may represent cooperativity between the two networks during the stage where these associative and executive processes are most engaged (looking at the stimulus and generating novel uses).

Activity between the DMN and FPN during mind wandering was more diverse, with theta activity differing significantly between the two networks during both the stimulus presentation stage and response stage, as opposed to its dynamics during divergent thinking. The current body of evidence strongly suggests that the DMN plays a central role in supporting mind wandering.^9,63^ Recent studies have implicated the role of FPN functional connectivity with other networks in mind wandering, such as its interaction with the visual network.^64^ The difference in theta band activity may indicate that the DMN and the FPN are playing discrete roles from one another during mind wandering, reflecting their unique communications with other functional networks. Taken together, these findings speak to the complex pattern of activity occurring in the DMN and suggest that the AUT employed in this study might rely more on the interplay between DMN and other cognitive networks, while the mind wandering task engaged the DMN with more specificity.

### Task-dependent temporal evolution of DMN recruitment

When considering the initial DMN activity during the stimuli presentation stage, AUT displayed the strongest effect, characterized by low theta power and increased gamma activity. This was true not only when comparing AUT to mind wandering, but also when comparing it against the sustained attention task (ATT). Indeed, DMN activity during ATT was characterized by the opposite pattern with respect to AUT, showing high theta and low gamma. This result was obtained performing a control analysis employing the first 2 s of AUT and mind wandering to offer a time-matched comparison to the much shorter ATT task (Supplementary Fig. 2A). Looking across the entire time course revealed a dichotomy between the temporal dynamics of DMN activity in the AUT and mind wandering tasks. Specifically, the temporal evolution of DMN activity indicated that the alternate uses task engages the network in the early stage of the task (Fig. 3C and D, left), while participants were presented with an item and needed to covertly search through semantic and episodic information for alternative uses. During this stage, as discussed earlier, the FPN was also engaged. During the later response selection stage, theta was further suppressed, while gamma activity was increased (Fig. 3C and D, right). Our results align with fMRI research demonstrating DMN differential recruitment across different stages of the AUT^36,55,56,61^: this temporal evolution potentially reflects a more critical initial stimulus processing stage during AUT, for which the semantic information about the object needs to be searched, and ideas must be generated and subsequently compared to previous experiences to evaluate alternative uses for that object.^36,55^

In contrast, mind wandering displayed a weak response in the early task stage and a very strong effect during the later response production stage, when the participant was asked to recollect, out loud, their train of thought. The enhanced DMN recruitment during response production in the mind wandering task could reflect the increased memory demands of recalling and reporting a train of thought. Nonetheless, given previous findings^65,66^ of DMN involvement in mind wandering, the relatively lower DMN recruitment during the mind wandering stimulus period is surprising: the stimulus period of the mind wandering task is the phase during which the actual process of ‘mind wandering’ occurs—during the response window, the thoughts are merely reported, and this response more likely represents episodic memory-related DMN activation. However, during the stimulus period, mind wandering was not entirely spontaneous, as participants were also actively viewing a colourful shape—perhaps the stimulus acted as a ‘distraction’ from the mind wandering aspect, drawing the participants’ attention and making it harder for them to detach from the visual stimulus and allow for spontaneous thought to take over. Indeed, there is evidence that external sensory input can inhibit mind wandering.^67^ It is also noteworthy that most of the evidence on mind wandering arises from fMRI studies,^9^ thus slow fluctuations (<4 Hz, often <0.1 Hz), not examined here but known to modulate theta and gamma in DMN nodes,^22^ may offer a more direct correlate of the mind wandering process.^27^ Taken together, this evidence suggests that different cognitive functions elicit the DMN with their unique signatures of network activation, warranting further investigation to dissect the specificity of these function-signals relations.

Another significant implication of our result stems from the relative importance of the stimulus in the AUT versus mind wandering tasks: for the AUT, the stimulus played a critical role, guiding the semantic and episodic search for alternate uses.^36,68-70^ During the mind wandering task, on the other hand, the stimulus was just a probe intended to cue the participants to allow their minds to wander, with no requirement to perform any memory-guided processes. When the response window appeared, however, participants engaged in memory retrieval to report their train of thought.^36,71-73^ From this perspective, the DMN activity reported in our findings tracks the memory-intensive portions of the task, in line with current views that semantic and episodic memory systems are highly integrated with DMN activity.^74^ Our findings isolate the key role played by active recollection of memories as a driver of DMN activity, either as a covert search of alternative uses, or as overt retrieval of a previous train of thoughts.

### Spatial distribution of theta and gamma modulation within the DMN

Thus far, we have discussed the dynamics of DMN activity as a whole but the effects we observed differed across regions of the DMN. In particular, theta effects displayed a complex three-way interaction between tasks, their stages and DMN subnetworks: dorsomedial DMN theta exhibited the greatest dissociation between the task stages of the mind wandering task, while no clear subnetwork difference was evident in AUT. Meanwhile, gamma modulation was clearly driven by lateral DMN, with a similar pattern across the mind wandering and AUT (increasing from the stimulus to the response task stages for both; Fig. 4A).

To assess the spatio-temporal features of theta and gamma more holistically, we decided to exploit the temporal evolution of the signals without the artificial separation in two stages, revealing their associations using similarity-based analysis (Fig. 4B). We demonstrate that theta power differentiates the thought processes underlying the mind wandering versus AUT, displaying stronger modulation during mind wandering. Thus, theta power dynamics featured strong dissimilarities between our tasks. The similarity analysis results and the three-way interaction results discussed earlier (Fig. 4A and B) revealed relatively lower theta activity in lateral temporal cortex versus dorsomedial DMN early in the time course of the mind wandering task.^55^ Dorsomedial DMN theta suppression was more dramatic but delayed, occurring during the response stage and coinciding with increased gamma power in lateral DMN. The engagement of dorsomedial areas (PCC, SFG, mPFC) during the recollection stage of the mind wandering process could be reflecting memory retrieval, as theta-phase synchronization between posterior medial cortex and non-DMN regions has been previously implicated in autobiographical judgements.^20^

Gamma-band signals, classically considered to be reflecting local neural activity, were distinct between the DMN subsystems, but patterns of activation were more similar across the tasks: lateral structures were the major contributors to the observed gamma power effects for both AUT and mind wandering tasks (Fig. 4). Our findings demonstrate that theta and gamma signatures provide non-redundant information about the ongoing DMN activity: theta is modulated differently according to the cognitive processes at play, while gamma is recruited with a strong spatial gradient biased toward lateral temporal cortex for both processes. The steady contribution of lateral temporal gamma activity across our tasks is congruent with this region's implication in inner speech and semantic processing.^74^ Indeed, the proximity of default mode and language areas within the lateral temporal cortex is highly suggestive of multiple adjacent distributed networks that work closely to support different facets of the same higher cognitive function,^75^ like the ability to construct and maintain an internal narrative, continuously ongoing during mind wandering. Semantic processing and category-specific memory, which are key components of the AUT, have been historically localized to the MTG, a subregion of the lateral DMN.^76^ Furthermore, the MTG has been associated with the ability to attribute an action (use) to an object,^77^ a cognitive process certainly involved in the AUT. In fact, MTG seems particularly attuned to categories related to ‘tools’,^78^ matching our AUT stimuli. Beyond internal speech and semantic processing, the lateral temporal cortex is also important for memory. Closed-loop stimulation of the lateral temporal cortex has been employed to enhance episodic memory,^79^ implying that memory performance does not rely uniquely on the classic medial temporal network, but that lateral DMN subnetworks make distinct and behaviourally relevant contributions. By considering our results in this context, we corroborate the modern view of the DMN as a heterogenous and multiplexed system, composed of anatomo-functional subnetworks subserving different cognitive functions.^80^

### Causal relationship between DMN activity and divergent thinking

Creativity is a multifaceted concept that can have multiple meanings in different contexts.^81,82^ For divergent thinking, as tested by the AUT, the creativity of responses can be quantified in several ways.^83,84^ For instance, novel, surprising responses that are very distinct from the prototypical use of the cue object would score high in originality. On the other hand, fluency refers to the ability to produce a large number of responses. However, as fluency does not traditionally consider the identity of the responses, if a subject generates many nearly identical responses, the fluency score will be high. Thus, we designed an alternative measure to better reflect a participant's ability to ‘fluently’ produce a variety of responses. This metric, called ‘variability’, is similar to fluency in that it is higher for a larger number of responses; but it also takes into account how distant each response is from all the other responses. As a result, similar responses are less strongly weighted than distinct responses. Finally, another variation of fluency is flexibility—or the number of semantic categories of the responses. Like variability, flexibility also downweights similar responses and amplifies distinct responses. However, it does so in an ‘all-or-nothing’ manner: all responses that fall within the same semantic category are treated as a single response. Having defined these aspects of creativity, we asked whether the DMN is involved in any of them; if so, does the DMN play an equal role in the different facets of creativity, or do originality and fluency differentially depend on DMN activity?

Previous work using direct brain stimulation suggests that dominant, lateral DMN nodes contribute to creative fluency, but not originality.^29^ Yet, in the current study, disrupting DMN activity with high frequency stimulation reduced the originality of the responses but had no effect on fluency, variability or flexibility of the content. In other words, stimulation specifically modulated how original the alternative uses produced were, without affecting the variability across responses or number of semantic categories used (Fig. 5). There were notable differences in the behavioural scoring choices, experimental protocol (intraoperative), stimulation parameters (cycles of 10 s versus 3 s in the present study) and locations that could explain these different findings. Shofty *et al*.^29^ only included responses that were substantially different from the common use of each object, whereas the current study counted all responses. For the originality measure, responses other than the traditional use of the object were also excluded if they were frequently produced, to avoid inflating the fluency. In the current study, DMN stimulation occurred in different locations across subjects; either medial or dorsal PFC and, for one subject only, in lateral temporal cortex. In contrast, Shofty *et al*.^29^ stimulated the lateral dominant DMN exclusively. The partial effect on divergent thinking reported here (as well as the partial, though different, effect in Shofty *et al*.^29^) may therefore be related to the different anatomical target (see Weinberger *et al*.^85^ and Chen *et al*.^86^ for a review of non-invasive stimulation studies). Based on our electrophysiology results, the lateral DMN seems to be a more critical hub, as this subsystem was the main driver of local neural activity (gamma) for AUT (as well as mind wandering). This interpretation aligns well with existing literature about the effect of stimulation on memory and free recall: for instance, Ezzyat *et al*.^79^ demonstrated that stimulation in the lateral temporal region of the DMN was able to robustly modify memory performance.^79^ It is possible that the effect on originality reported in our study reflects the reduced ability to access memories related to the objects, consequently affecting the ability to generate original and non-canonical responses, without interfering with other aspects. More research is needed to disentangle the causal contribution of different DMN subsystems to the widespread set of cognitive processes that underlie divergent thinking.

Besides the AUT, we also examined the effect of DMN stimulation on mind wandering. The trains of thought reported in the mind wandering task were unaffected by stimulation, suggesting that divergent thinking depends more strongly on DMN integrity than does mind wandering. Our results indicate that the thought processes occurring during daydreaming and mind wandering are more resilient to external perturbation of the system. It is possible that changes in the train of thoughts might be more difficult to detect given their ‘unconstrained’ fluctuating nature, also in line with the subjective experience of a constant and coherent internal narrative. In addition, we scored the mind wandering responses using a similar approach to the one used for AUT, while it is possible that the cognitive process that occurred during our mind wandering task could not be appropriately measured through semantic distance metrics. Another possible explanation for the lack of perturbation effects on mind wandering originates from our electrophysiology findings: mind wandering displayed a complex interaction between task stages and theta activity across the two DMN subsystems. Theta is typically regarded as a long-range communication channel; therefore, to affect mind wandering, it might be necessary to tap into lower frequency communication channels with patterned stimulation (while the current study used high frequency stimulation) and potentially stimulate several DMN nodes simultaneously.

### Limitations and future directions

A limitation of our experimental design is our inability to time-lock task events with internal thought processes. In an attempt to constrain the trial time course to allow for accurate analysis of the associated cognitive functions, we split trials into stimulus and response stages. However, due to the long duration of the trial structure, we notice that it may be impossible to fully disentangle cognitive processes corresponding to stimulus versus response: once participants begin to understand the structure of the task, especially for AUT, they may begin to ‘prepare’ their responses even before the stimulus stage is complete. However, the differential activation seen, e.g. during the mind wandering response window and not during the stimulus window, suggests that robust network activation takes place during the latter timeframe despite potential preparatory effects.

Although standard for human invasive experiments, our study is based on a small sample size (*n* = 13). To limit potential bias, we employed within-subject approaches for all our analyses, and all group-level analysis are based on the consistency of within-subject differences. By nature of the technique employed we were also limited to clinically-defined electrode trajectories, constraining our ability to equally sample within and across individual cortical areas. For example, the PCC, a key DMN hub was rarely sampled in our cohort. Consequently, our subdivision in dorsomedial and lateral DMN is an oversimplification of the network and its subsystems, arising in part from previous evidence and in part by our cortical sampling availability. In particular, our dorsomedial-DMN system spanned medial and dorsal frontal areas, posterior medial cortex and parietal locations, potentially mixing together in one subsystem several unique contributions. This said, our study was not aimed at dissecting the individual contribution of each DMN node, and whole-brain techniques are better suited to inform on the precise organization of largely distributed subnetworks.

There are very few studies employing direct cortical stimulation of DMN regions during active behaviour.^28,29,87^ In general, stimulation experiments are particularly challenging and require the consideration of several clinical factors, often constraining the ability to perform rigorous controls (i.e. selecting consistently the same stimulation site across individuals, repeating the experiment with a control stimulation site outside the network or sham condition). In this study, we were unable to select posterior medial cortical locations as the stimulation sites, preventing us from drawing direct comparisons with previous studies targeting PCC. Our results demonstrate that the originality of ideas can be diminished by interfering with different nodes of the DMN. There is a need to understand how different stimulation parameters entrain neural activity (both locally and distally) and how this relates to effects on behaviour. More studies will be required to identify the necessary intra- and inter-network communication mechanisms and how these relate to findings of juxtaposed neural populations with episodic and executive functional profiles.^88^ Indeed, creativity and divergent thinking have also been shown to involve cognitive control networks, such as the executive control network.^59,89-91^ For example, studies have demonstrated the significance of key brain regions associated with top-down control, such as frontoparietal system, in the performance of a variety of cognitive tasks linked to creativity.^92,93^ Divergent thinking seems to be subsisted by multiple brain networks, such as the executive and the salience network, and their synchronization with the DMN could support the generation of novel, divergent thoughts.^32,33,55,56^ While our study supports this interpretation, especially considering the similar activity between DMN and FPN during the stimulus stage of AUT, our analyses did not investigate inter-network communication. To better quantify the relationship between these various networks, future studies focused on measures of network synchrony, such as cross-frequency coupling and phase synchronization, will be necessary.^94^ This will allow for further insight into how the DMN may coordinate with and integrate activity from other functional networks across a range of spatial and temporal scales. Overall, our results provide unique and causal insights into the role of the DMN in complex cognitive processes.

## Supplementary Material

awae199_Supplementary_Data

## Data Availability

The data that support the findings of this study are available on request from the corresponding author.
