# Supplementary material for: Default mode network electrophysiological dynamics and causal role in creative thinking
Source: Brain. 2024 Jun 18;147(10):3409–25. doi: 10.1093/brain/awae199 (PMC11449134; doi:10.1093/brain/awae199)
Supplement: awae199_Supplementary_Data [file awae199_supplementary_data.pdf]

Supplementary Material:
Default mode network electrophysiological dynamics and causal role in creative thinking

Eleonora Bartoli[1,†], Ethan Devara[1†], Huy Q. Dang[1], Rikki Rabinovich[2], Raissa K. Mathura[1], Adrish Anand[1], Bailey R. Pascuzzi[1], Joshua Adkinson[1], Yoed N. Kenett[3], Kelly R. Bijanki[1,4], Sameer A. Sheth[1,4] and Ben Shofty[2]

[†]These authors contributed equally to this work.

[1]Department of Neurosurgery, Baylor College of Medicine, USA
[2]Department of Neurosurgery, Clinical Neuroscience Center, University of Utah, USA
[3]Faculty of Data and Decision Sciences, Technion – Israel Institute of Technology, Israel
[4]Department of Neuroscience, Baylor College of Medicine, USA

1. Additional Sample information

1.1 List of antiepileptic medications

# SUBJECT	ANTIEPILEPTIC MEDICATIONS
**1**	Lacosamide 300mg, Oxcarbazepine 600 mg
**2**	Lacosamide 100mg, Oxcarbazepine 900mg
**3**	Lacosamide 100mg, Topiramate 150mg
**4**	Fosphenytoin 800mg, Lamotrigine 100mg, Oxcarbazepine 1200mg, Topiramate 25mg
**5**	Lamotrigine 200mg, Topiramate 100mg
**6**	Lamotrigine 300mg, Levetiracetam 1500mg
**7**	Cannabidiol 200mg, Lamotrigine 300mg, Levetiracetam 1250mg
**8**	Lamotrigine 200mg, Perampanel 4mg
**9**	Levetiractam 2000mg
**10**	Levetiractam 2000mg, Rufinamide 1600mg, Zonisamide 200mg
**11**	Lamotrigine 200mg, Zonisamide 500mg
**12**	Brivaracetam 100mg, Cannabidiol 2.5mg/kg, Lacosamide 200mg, Lamotrigine 150mg
**13**	Clobazam 10mg, Lamotrigine 300mg

Table S1. The list of antiepileptic medications for each patient.

1.2 Cortical coverage for each individual subject: electrode classification and selection

In this study, we classified our electrodes using the 7 Network atlas, which identifies functionally coupled regions via resting-state based cortical parcellation obtained from a normative sample of 1000 healthy individuals, (Yeo et al., 2011). Figure S1 displays the 7 Network atlas on a template brain (Freesurfer Colin27, upper left panel), as well as the legend and color scheme for each of the 7 Networks. The template brain is displayed using three views: medial view of the right hemisphere (hiding the left hemisphere), frontal view of both hemispheres, lateral view of the right hemisphere. The rest of the panels show the cortical surface for each of the patients that participated in our study (13 subjects, denoted as S1 to S13) as a semi-transparent surface. For each individual panel, the top row displays all electrodes and their classification based on the 7 Network atlas; the bottom part displays the electrodes included in the analyses (red = Default; orange = Fronto-parietal; white = Default network stimulation location, if performed), following the exclusion criteria described in methods (based on recording noise, epileptic onset zone, proximity to other networks, etc.). Note that in one view (i.e., right side of all panels) the left hemisphere is hidden, but all the electrodes are displayed (both left and right). The right hemisphere is shown for reference.

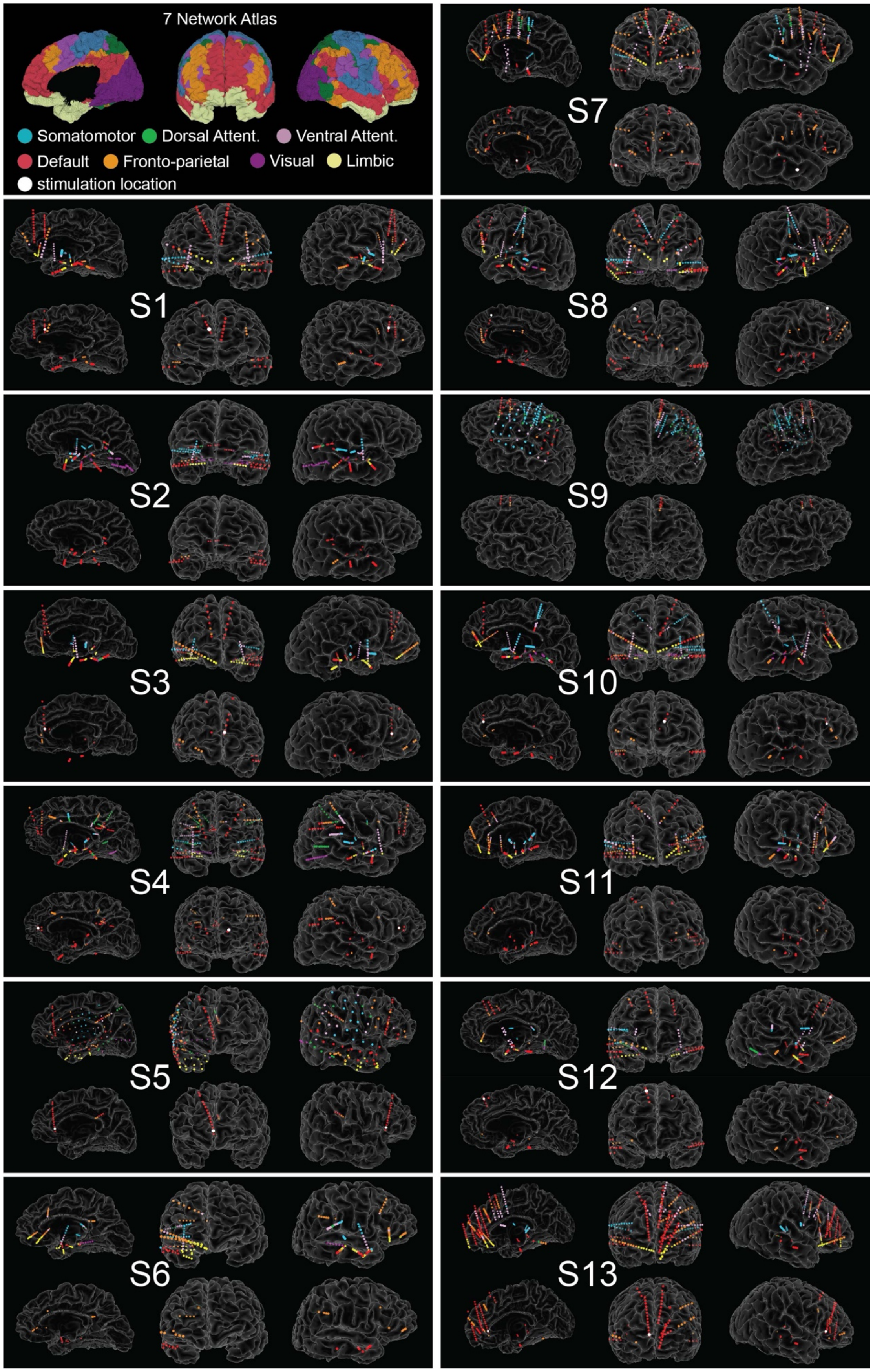

Figure S1. Classification of electrodes based on their proximity to the 7 Network atlas. The top left panel shows the 7 Network atlas on a standard brain (Colin27). For each subject (S1 to S13), the top row shows all the electrode contacts and their classification according to the 7 Network Atlas; the bottom images show the electrodes used for analysis, after applying exclusions due to noise or proximity to other networks. DMN: red; FPN: orange; stimulation contact: white.

2. Control analyses

2.1 DMN versus FPN analyses: comparison of results obtained using different statistical approaches.

In the main manuscript, we employed multi-level mixed effects models: these models are appropriate for nested and unbalanced data structure (multiple electrodes for each subject, with varying numbers) and do not require information loss (averaging across electrodes). The statistical significance of different fixed effect variables of interest (e.g., Network) was evaluated by comparing models with/without the effect of interest (e.g., a model with and without the fixed effect of Network, all other components the same). A significant difference arising in this model comparison indicates that the overall addition of that fixed effect (complex model) increases the ability of the model to capture the data (weighted against the cost of the additional coefficients). This approach captures the overall impact of including the fixed effect of interest in the model (i.e., main effect and interaction terms together) rather than the main effect alone. In addition, in the main manuscript analyses, we reported post-hoc comparisons on the model results (using z-statistic approximation). Here, we include a comparison to a more classic approach: we averaged all the observations from a subject (i.e., averaging across electrodes) and used a repeated-measures ANOVA to test for main effects and interactions.

Rm-ANOVA: the dataset was averaged to obtain only one averaged value per subject and per network rather than multiple electrodes for each subject. The averaged dataset was analyzed using repeated measures ANOVA with Subject as the pairing variable and Network (DMN, FPN), Task Stage (Stimulus, Response), and TimeBin (29 values, from 0.5 to 15 seconds, continuous) as fixed effects, modeling all main effects plus their interaction terms.

MEM: mixed effect model results (original dataset, not averaged across electrodes, modeled as nested random effects within each subject) from the main manuscript analyses were recomputed using Satterthwaite's method to increase comparability with the rm-ANOVA format.

Effect/Interaction terms	rm-ANOVA Sum of Squares	rm-ANOVA F	rm-ANOVA p-value	MEM Sum of Squares	MEM p-value		post-hoc comparisons: Network by Stage (p-adjusted, bonferroni, 4 comparisons)	rm-ANOVA posthoc results	MEM posthoc results	agreement
**MW Theta power**						post-hoc comparisons	Stimulus Stage: DM vs FP	p-adj = 0.0012	p-adj = 0.032	Y
**Network**	**20289**	**13.003**	**p<0.001**	**34086**	**p<0.001**					
Task Stage	254050	162.819	p<0.001	3785448	p<0.001		Response Stage: DM vs FP	p-adj <0.001	p-adj <0.001	Y
TimeBin	8712	5.584	p = 0.018	202387	p = 0.013					
**Network * Task Stage**	**117976**	**75.61**	**p<0.001**	**557653**	**p<0.001**		DM: Stimulus vs Response	p-adj <0.001	p-adj <0.001	Y
Network * Time	607	0.389	p = 0.533	46794	p=0.003					
Task Stage * Time	2782	1.783	p = 0.182	17067	p = 0.078		FP: Stimulus vs Response	p-adj = 0.016	p-adj = 0.011	Y
Network * Task Stage * Time	586	0.375	p = 0.54	36539	p = 0.009					
**MW Gamma power**						post-hoc comparisons	Stimulus Stage: DM vs FP	p-adj = 0.015	p-adj = 0.97	N
**Network**	**5399**	**22.838**	**p<0.001**	**106**	**p = 0.69**					
Task Stage	37501	158.632	p<0.001	160516	p<0.001		Response Stage: DM vs FP	p-adj <0.001	p-adj <0.001	Y
TimeBin	884	3.741	p = 0.053	6691	p = 0.0018					
**Network * Task Stage**	**18687**	**79.047**	**p<0.001**	**68331**	**p<0.001**		DM: Stimulus vs Response	p-adj <0.001	p-adj <0.001	Y
Network * Time	282	1.194	p = 0.275	22208	p<0.001					
Task Stage * Time	5439	23.007	p<0.001	43691	p<0.001		FP: Stimulus vs Response	p-adj = 0.036	p-adj = 0.325	N
Network * Task Stage * Time	161	0.682	p = 0.41	14	p = 0.88					

Table S2. MW theta (top) and gamma (bottom) rmANOVA and MEM results compared (left) and post-hoc analysis for Network by Task comparisons (right).

The tables S2 and S3 display the results of the rm-ANOVA and MEM side by side (left side of each table) for the Mind Wandering (MW) and Alternate Uses (AUT) tasks, respectively. The main effect of Network and the interaction between Network and Task Stage are displayed in bold to highlight the result of interest for this control analysis (i.e., the terms that would contribute to the overall effect of Network reported in the main manuscript). Additionally, on the right side of each table, we report the outcome of the post-hoc tests obtained from each model, adjusted using Bonferroni (4 comparisons for each model) and their agreement (Y/N) in terms of overall significance. Note that the MEM post-hoc results are the values reported in the main manuscript and in Figure 3 (bar plot insets).

Effect/Interaction terms	rm-ANOVA Sum of Squares	rm-ANOVA F	rm-ANOVA p-value	MEM Sum of Squares	MEM p-value
**AUT Theta power**					
**Network**	**66906**	**41.164**	**p<0.001**	**58912**	**0.001675**
Task Stage	122174	75.168	p<0.001	2934166	p<0.001
TimeBin	40740	25.066	p<0.001	452578	p<0.001
**Network * Task Stage**	**32425**	**19.949**	**p<0.001**	**3328**	**p = 0.453148**
Network * Time	11920	7.334	p = 0.006	173669	p<0.001
Task Stage * Time	3866	2.379	p = 0.123	12822	p = 0.140897
Network * Task Stage * Time	5230	3.2218	p = 0.073	42775	p = 0.007161
**AUT Gamma power**					
**Network**	**139**	**0.43**	**p = 0.49**	**542**	**p = 0.30**
Task Stage	5578	18.554	p<0.001	286482	p<0.001
TimeBin	1063	3.537	p = 0.06	203	p = 0.52
**Network * Task Stage**	**9479**	**31.532**	**p<0.001**	**2248**	**p<0.001**
Network * Time	263	0.874	p = 0.35	502	p =0.32
Task Stage * Time	2132	7.092	p = 0.007	14940	p<0.001
Network * Task Stage * Time	20	0.065	p = 0.798	7927	p<0.001

	post-hoc comparisons: Network by Stage (p-adjusted, bonferroni, 4 comparisons)	rm-ANOVA posthoc results	MEM posthoc results	agreement
post-hoc comparisons (AUT Theta power)	Stimulus Stage: DM vs FP	p-adj = 0.67	p-adj = 1	Y
	Response Stage: DM vs FP	p-adj <0.001	p-adj = 0.04	Y
	FP: Stimulus vs Response	p-adj <0.001	p-adj = <0.001	Y
	DM: Stimulus vs Response	p-adj = 0.012	p-adj = 0.638	N
post-hoc comparisons (AUT Gamma power)	Stimulus Stage: DM vs FP	p-adj <0.001	p-adj = 1	N
	Response Stage: DM vs FP	p-adj = 0.002	p-adj = 0.003	Y
	DM: Stimulus vs Response	p-adj <0.001	p-adj <0.001	Y
	FP: Stimulus vs Response	p-adj = 1	p-adj = 0.144	Y

Table S3. AUT theta (top) and gamma (bottom) rmANOVA and MEM results compared (left) and post-hoc analysis for Network by Task comparisons (right).

Comparable results and overall interpretation based on post-hoc multiple comparisons.

While the significance of the individual terms varies slightly between the two approaches, the conclusions drawn from both approaches are similar, especially when considering the focus of the main manuscript analysis (overall importance of the fixed effect of Network and post-hoc comparisons testing for the differences between Network and Task Stage combinations). The only post-hoc discrepancy between the two statistical methods was a significant difference between networks during the stimulus stage derived from the rm-ANOVA but not detected from the Mixed Effects model. The rm-ANOVA, relying on averaged data, does not have information about inter-electrode variability, leading to potentially biased estimates with respect to MEM (where this source of variability is part of the model as a random effect). We performed this comparison as a general proof of concept that the results presented in the main manuscript using mixed effect models and sequential model comparisons are robust and incorporate the variability introduced by random effects. In any case, comparable findings would be reported using a different approach.

2.2 *DMN engagement across all tasks.*

We compared the neural signatures of the DMN occurring during the start of the three different tasks: AUT, MW, and sustained attention (ATT). By nature of the sustained attention task, the duration of each ATT trial was much shorter than the time windows used in the AUT or MW. To avoid confounds related to the different temporal duration of the tasks, we focused on the first two seconds of each task, aiming to capture DMN activity during the entire ATT trial as well as the initial DMN engagement of the DMN during the early encoding of the object (AUT) or shape (MW). Thus, we examined theta and gamma power values computed over the first 2 seconds from stimulus onset, employing a sliding 1 s window with 50% overlap (Fig. S2A). We used a linear mixed effects model for both frequency ranges (theta and gamma) to assess variations in band power as a function of the task type (ATT, AUT, MW: fixed effect; subject and electrodes: nested random effects). We then performed two further control analyses. Our first control analysis ensured that we did not include button presses when analyzing ATT data. To this end, we repeated the above analysis, but optimized the analysis time-window for each patient. Rather than just considering the first 2 seconds of the trial, we determined the duration of the shortest ATT trial for each patient and selected this duration as the time window for that patient. In other words, for each patient, we computed average theta and gamma power over this period between stimulus onset and the end of the shortest ATT trial. We designed a second control analysis to evaluate the specificity of DMN engagement with respect to a different functional network (i.e., somatomotor network, not expected to show any particular engagement in MW or AUT). Consequently, we analyzed gamma and theta activity using electrodes localized in the somatomotor network.

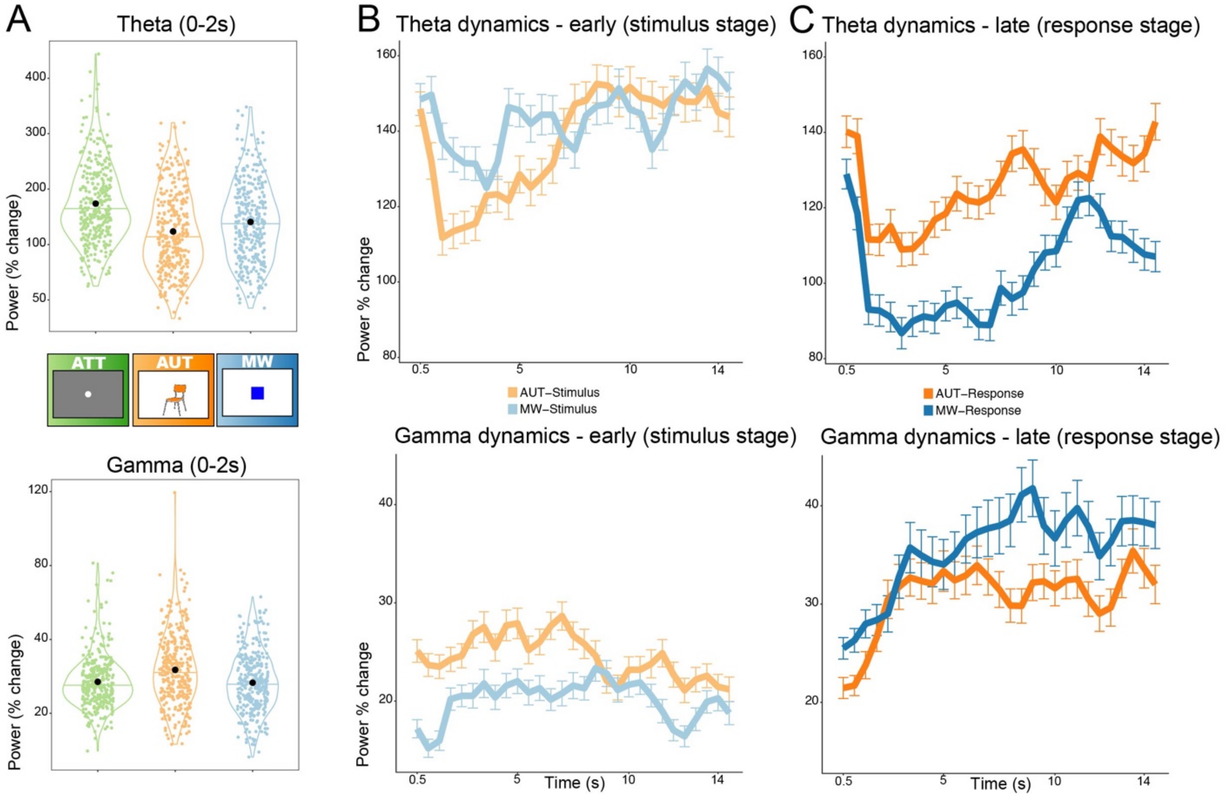

Figure S2. Engagement of DMN activity during the tasks. Panel A: Theta (4-8Hz, top) and gamma (30-70Hz, bottom) power modulations recorded in DMN during the first 2 seconds from stimulus onset for each task, as percent change from pre-stimulus baseline. All tasks increased theta and gamma power above baseline. Comparing the tasks, theta power was highest during attention (ATT) followed by mind wandering (MW) and was lowest for creativity (AUT), all comparisons *p*-adj < .01. Gamma power was modulated in the opposite way, being largest for AUT and with no differences between MW and ATT. Each point in the violin plots represents an electrode in the DMN (n=333). Panel B: temporal evolution of theta and gamma activity during creativity (AUT, orange) and mind wandering (MW, blue) from stimulus onset (lighter colors) and from response prompt (darker colors). The dynamics reveal how the DMN is engaged differently according to the task stage: during the first 6-8 seconds of viewing the stimuli, theta and gamma are more strongly modulated by AUT than MW. In the response stage, the opposite pattern emerges, with lower theta and greater gamma increases occurring during the verbalization of the train of thoughts in MW.

DMN engagement at stimulus onset displays opposite theta and gamma modulations.
Power in the theta and gamma frequency bands in the DMN was differentially modulated during the viewing of the stimuli for the three different tasks: alternate uses task (AUT), mind wandering (MW), and sustained attention (ATT) (Fig. S2A). Both DMN theta and gamma were significantly modulated by the task type (theta model comparison without/with task type as a fixed effect: $\chi^2(2) = 89.04$, $p < .001$; gamma: $\chi^2(2) = 38.45$, $p < .001$). As a whole, low and high-frequency power both increased during all three tasks. However, the AUT and MW tasks were characterized by lower theta power compared to the ATT task (mean ± standard error computed across subjects and electrodes; ATT: 173.8±3.9%; AUT: 123.6±4.01%; MW: 140.7±3.9%). Pairwise post-hoc comparisons confirmed that theta power differed between all three tasks, with the alternate uses task showing the lowest theta within the DMN (AUT vs. ATT: z = -9.6, *p*-adj < .001; MW vs. ATT: z = -6.3, *p*-adj < .001; MW vs. AUT: z = 3.3, *p*-adj = .003).

In the gamma range, the opposite pattern emerged, with the AUT inducing the largest increase in gamma power; MW and ATT both yielded relatively lower increases in gamma power, and there was no significant difference between these two tasks (ATT: 17.1±0.79%; AUT: 23.5± 1.04%; MW: 16.6±0.87%; AUT vs. ATT: z = 5.2, *p*-adj < .001; MW vs. ATT: z = -0.4, *p*-adj = 1; MW vs. AUT: z = -5.6, *p*-adj < .001). The same analysis was repeated using a shorter time window (based on the ATT trial length), yielding virtually identical results. Overall, by contrasting the initial stage of DMN tasks versus attention, we report that DMN engagement features low theta power for both AUT and MW, coupled with an high gamma-range activity specifically for the alternate uses task (Fig. S2A).

As a control (described earlier), we repeated the above analysis on data collected from electrodes in a different network (somatomotor network, specifically pre-central gyrus). The theta activity pattern in the somatomotor network showed a task-related modulation different from the one found in the DMN. Specifically, theta power only increased for the sustained attention task, and no differences were present between the default mode tasks (model comparison with/without the fixed effect of task: $\chi^2(2) = 15.8$, $p < .001$; AUT vs. ATT: z = -4.2, *p*-adj < .001; MW vs. ATT: z = -2.2, *p*-adj = .08; MW vs. AUT: z = 1.99, *p*-adj = .14). Gamma activity in the somatomotor network was not modulated by any of the tasks, and no differences between tasks were found (adding the fixed effect of task type did not significantly improve the model, $\chi^2(2) = 0.38$, $p = .8$; all post-hoc comparisons between tasks not significant). Thus, the aforementioned differences in DMN theta and gamma activity during mind wandering and alternate uses task stimuli are not general brain-wide patterns, since they do not extend to the somatomotor network. From these findings, we can conclude that alternate uses and mind wandering tasks successfully engaged DMN, compared to a control task (ATT) and a non-DMN network (somatomotor).

DMN dynamics during MW and AUT Tasks.
Figure S2B and C offer a visual display of the different dynamics between MW and AUT. These panels are a re-organization of the same panels in Figure 3 without the FPN for an easier visualization of the differences between MW and AUT during the task stages and over time, reported in the main text (*DMN dynamics result section*).

2.3 Stimulation effect on behavior when excluding Subject #1

We evaluated the behavioral data from the subjects that received stimulation with the same stimulation frequency settings (130 Hz; n = 8) by excluding the first patient (Subject #1, stimulated using 50 Hz). We repeated the main manuscript analyses and compared the originality scores for stimulation versus non-stimulation trials in this subset of patients.

High-frequency stimulation of electrodes within the DMN reduces originality of alternate uses responses when excluding Subject #1.

Alternate Uses: after excluding Subject #1, we still found that stimulation reduced originality in AUT (median SemDis without stimulation: 0.97; with stimulation: 0.95; W = 60, p = .0018). These results were virtually identical to the ones reported in the main manuscript (obtained considering n = 9 subjects, including Subject #1).

2.4 Stimulation effect on behavior at a trial level

In addition to analyses described in the main manuscript, we also investigated the effect of stimulation of originality at the trial level. In this analysis, we considered all trials (rather than averaging the scores across trials for each subject) across all participants (n=13), including the 4 subjects that did not receive stimulation due to experimental constraints. Specifically, we used non-parametric statistical tests to compare trials with stimulation versus trials without stimulation (Mann-Whitney Test, independent samples). This analysis served as a control to ensure that the difference between stimulation and no stimulation trials could be reliably detected even when considering all subjects (and not only as a within-subject effect).

High-frequency stimulation of electrodes within the DMN reduces originality of AUT responses

Including trials from subjects that did not receive stimulation (n = 4, median SemDis: 0.97) and testing for differences between all stimulated and non-stimulated trials in the full sample confirmed the reduction in AUT originality scores (n = 13; W = 2638.5, p = .017). This finding supports the robustness of the results reported in the main text.

2.5 *Frequency bands*

In the manuscript, we selected theta (4-8 Hz) and gamma (30-70 Hz) band signals as the signals of interest. We based our selection on the large body of literature on gamma power as indexing local neural activity and theta as a larger-scale signal modulated by memory processes and DM functions. Here, we employed a data-driven approach to evaluate the association between these two signals and other classic signals occupying different frequency ranges. We computed power changes (with respect to baseline) in delta (2-4 Hz), alpha (8-12 Hz), beta (12-30 Hz) and high-gamma (70-150 Hz) to complement our signals of interest. We performed PCA using scaled versions of all the signals (delta, theta, alpha, beta, gamma and high-gamma) as features, with the observations being the normalized power values at each time-bin for each, response stage, task, electrode and subject in our dataset. By examining the similarity between the original features (loadings over the first two dimensions, depicted in the biplot in Fig. S3), we note that the other frequency bands do not seem to capture additional variability with respect to gamma and theta (gamma and high gamma loadings are very similar to each other; low frequency band signals are similar to theta). This data-driven approach validates the selection of theta and gamma as the signals capturing most of the non-redundant variability in our dataset.

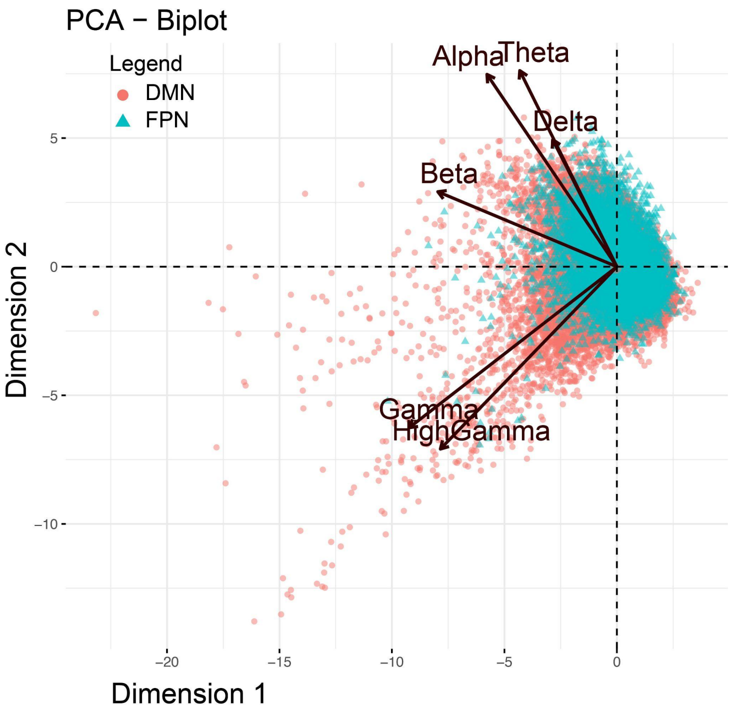

Figure S3. Association between different neural signals in our dataset. Biplot showing the observations projected on the first two components (dimension 1 and 2) and the projection of the original features (loadings) as arrows. Gamma (30-70Hz) and theta (4-8 Hz) are associated with the first and second component while being orthogonal to each other. The other low-frequency range signals (delta, alpha and to a certain degree beta) are closely associated with theta (small angle between their projections), while high-gamma is closely associated with gamma.
